# Genome-Wide Characterization and Salt-Responsive Expression Divergence of Chromosome Group 2 and Group 6 *TaBADH* Genes in Wheat

**DOI:** 10.3390/plants15172577

**Published:** 2026-08-24

**Authors:** Hua Li, Mengxue Huang, Shuxin Zhang, Xiaoyu Yang, Lingyu Pan, Sitong Wang, Wanjun Yang, Hongtu Qiu, Yemeng Zhang, Chunwang Jia, Xiu Yang

**Affiliations:** 1 School of Life Science and Engineering, Jining University, No. 1 Xingtan Road, Qufu 273155, China; 17263357330@163.com (H.L.); yangxiu022619@163.com (H.Q.); zhangyemeng@jnxy.edu.cn (Y.Z.); 2 Experimental Teaching and Equipment Management Center, Jining University, No. 1 Xingtan Road, Qufu 273155, China; 3 Jiangsu Xianchun Agricultural Development Company Limited, Nanjing 211113, China; 18281688408@163.com; 4 Department of Art and Design, Jining Polytechnic, No. 77 Jinyu Road, Jining 272037, China

**Keywords:** betaine aldehyde dehydrogenase, bread wheat, glycine betaine, homeolog, salt stress, *TaBADH-6D*

## Abstract

Betaine aldehyde dehydrogenase (BADH) catalyzes the final step in glycine betaine biosynthesis, but the evolutionary divergence and differential salt responsiveness of *BADH* homeologs in bread wheat remain unclear. We identified six *TaBADH* genes and analyzed their phylogenetic relationships, conserved motifs, gene structures, promoter cis-acting elements and synteny. RNA-seq and qRT-PCR were used to compare expression in salt-tolerant Jimai 60 and salt-sensitive Chinese Spring under 200 mM NaCl, and BADH activity, glycine betaine, H_2_O_2_ and malondialdehyde (MDA) were measured during treatment. The genes separated into chromosome group 2 and group 6 clades with distinct structural and transcriptional patterns. *TaBADH-2B* encoded a shorter protein and lacked several conserved motifs. Group 6 genes showed stronger salt-responsive expression in Jimai 60, with *TaBADH-6D* displaying the strongest and most sustained induction. Jimai 60 also showed higher BADH activity and glycine betaine accumulation and lower H_2_O_2_ and MDA contents at later time points. Expression of *TaBADH-6D* improved *E. coli* growth under 200 mM NaCl. These findings identify homeolog-specific divergence within the *BADH* wheat family and support *TaBADH-6D* as a candidate for plant-level functional validation.

## 1. Introduction

Soil salinization affects approximately 1.381 billion hectares of land worldwide (10.7% of the global land surface) and up to 10% of both irrigated and rainfed croplands [1,2]; in China, 36.7 million hectares are salt-affected, of which 12.3 million hectares retain agricultural potential [3]. Although bread wheat (*Triticum aestivum* L.) supplies 20% of the calories and protein consumed worldwide, it exhibits only moderate salt tolerance; improving its performance on saline soils is therefore important for maintaining yield stability [4,5,6]. At the cellular level, plant adaptation to salt stress depends largely on the capacity to preserve osmotic balance, protect macromolecules and sustain metabolic homeostasis [7], with the accumulation of compatible solutes representing a major protective strategy [8].

Glycine betaine is a major compatible solute involved in plant responses to hyperosmotic stress, and the final step in its biosynthesis involves the NAD^+^-dependent oxidation of betaine aldehyde to glycine betaine by betaine aldehyde dehydrogenase (BADH), an enzyme of the ALDH10 family [9,10,11,12]. Heterologous expression or overexpression of *BADH* genes from diverse halophytes and glycophytes has been reported to enhance stress tolerance in *Arabidopsis*, tomato, potato, tobacco, soybean, maize, kiwifruit and wheat [13,14,15,16,17,18,19,20]. These studies support conserved stress-related functions of BADH enzymes and their potential utility for improving salt tolerance in crops. Although wheat naturally accumulates glycine betaine [21], previous studies have focused on individual wheat *BADH* loci, leaving differences between homeologs in the A, B, and D subgenomes unresolved. Sun et al. (2019) [17] characterized a *BADH* gene located on chromosome 6AL and showed that its overexpression enhanced salt tolerance in Arabidopsis. Yu et al. (2022) [22] identified an intronic indel in *TaBADH-A1* associated with variation in drought and salt responses. These studies linked individual *BADH* loci to abiotic stress responses but did not assess the complete gene family across all three wheat subgenomes. Although a genome-wide analysis of *BADH* genes has been reported in maize [23], no comparable homeolog-resolved analysis has been conducted in bread wheat. Given wheat’s allohexaploid genome, comparative analysis of the structural and transcriptional characteristics of *BADH* homeologs may help identify family members associated with salt responses [24,25,26].

In this study, we aimed to: (1) characterize the evolutionary relationships, chromosomal distribution, conserved motifs, gene structures, promoter cis-acting elements, and syntenic relationships of wheat *BADH* homeologs located on chromosome groups 2 and 6; (2) characterize their salt-responsive expression patterns in Chinese Spring and Jimai 60 and examine the relationships between these patterns and changes in BADH activity, glycine betaine accumulation, and oxidative-damage indicators under NaCl treatment; and (3) identify the most consistently salt-responsive *TaBADH* member and preliminarily assess the growth phenotype associated with its heterologous expression in *Escherichia coli* under NaCl stress.

## 2. Results

### 2.1. Identification and Physicochemical Characterization of TaBADH Family Members

Six *TaBADH* family members were identified using HMMER 3.0 and confirmed by InterPro domain analysis (Table 1). The six predicted proteins ranged from 311 to 506 amino acids in length, with molecular weights of 32.6–54.6 kDa and theoretical isoelectric points of 5.06–5.88. Notably, the protein encoded by *TaBADH-2B* was markedly shorter (311 aa) than those encoded by the other five genes (502–506 aa). Instability indices ranged from 31.1 to 35.7, and aliphatic indices from 88.5 to 99.9. The grand average of hydropathicity (GRAVY) values differed between chromosome groups: the chromosome group 6 members, TaBADH-6A, TaBADH-6B, and TaBADH-6D, had negative values (−0.074 to −0.066), whereas the chromosome group 2 members, TaBADH-2A, TaBADH-2B, and TaBADH-2D, had positive values (0.028 to 0.264). All six proteins were predicted to localize to the chloroplast, cytoplasm, and peroxisome (Table 1).

### 2.2. Phylogenetic Relationships and Chromosomal Distribution of TaBADH Genes

**Figure 1:**
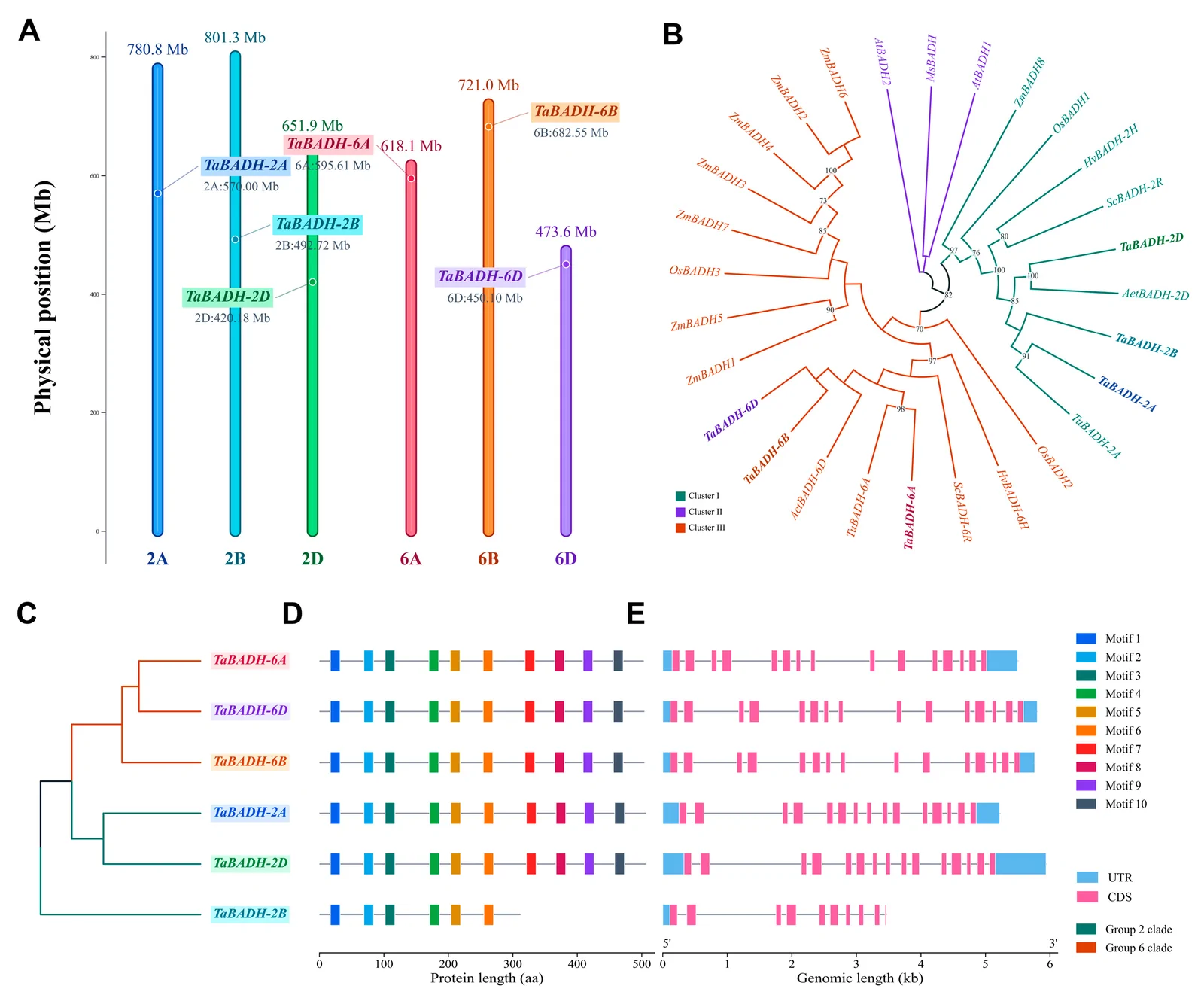
Chromosomal distribution, phylogenetic relationships, conserved motifs and gene structures of *TaBADH* genes. (**A**) Physical locations of the six *TaBADH* genes on wheat chromosomes. (**B**) Maximum-likelihood phylogenetic tree of BADH proteins from wheat, rice, maize, *Arabidopsis thaliana*, *Medicago sativa* and representative Triticeae species. Bootstrap values ≥ 70% are shown at the corresponding nodes, and different colors indicate distinct *BADH* clusters. (**C**) Maximum-likelihood tree of the six TaBADH proteins. (**D**) Distribution of ten conserved motifs. (**E**) Exon–intron structures of the six *TaBADH* genes.

The six *TaBADH* genes were located on chromosomes 2A, 2B, 2D, 6A, 6B and 6D (Figure 1A). Phylogenetic analysis of BADH proteins from wheat, rice, maize, *Arabidopsis thaliana*, *Medicago sativa* and four representative Triticeae species resolved the proteins into three clusters (Figure 1B). Bootstrap values ≥ 70% are shown at the corresponding nodes. TaBADH-2A, TaBADH-2B, and TaBADH-2D clustered with BADH proteins from the four Triticeae species, OsBADH1, and ZmBADH8 in Cluster I. TaBADH-6A, TaBADH-6B, and TaBADH-6D clustered with BADH proteins from the four Triticeae species, OsBADH2, OsBADH3, and several ZmBADH proteins in Cluster III. Cluster II comprised AtBADH1, AtBADH2, and MsBADH.

### 2.3. Phylogeny, Conserved Motifs and Gene Structures of the TaBADH Family

To examine the evolutionary relationships among *TaBADH* family members, a maximum-likelihood tree was constructed using the full-length amino acid sequences of the six TaBADH proteins, with 1000 bootstrap replicates (Figure 1C). The six TaBADH proteins formed two clades corresponding to their chromosome groups: the group 6 clade comprised TaBADH-6A, TaBADH-6B, and TaBADH-6D, whereas the group 2 clade comprised TaBADH-2A, TaBADH-2B, and TaBADH-2D. A total of 10 conserved motifs (motifs 1–10) were identified across the six TaBADH proteins using MEME (Figure 1D). Motifs 1–6 were present in all six proteins and occurred in the same order along their sequences, whereas motifs 7–10 were absent from TaBADH-2B but present in the other five proteins. Exon–intron analysis (Figure 1E) showed that the five genes encoding the longer proteins shared similar architectures, with genomic lengths of approximately 5–6 kb and comparable exon numbers and positions; by contrast, *TaBADH-2B* spanned approximately 3.5 kb and contained fewer exons. The conserved-motif composition and exon–intron architecture were consistent with the phylogenetic separation of the *TaBADH* family into group 6 and group 2 clades, whereas *TaBADH-2B* differed structurally from the other group 2 members. Multiple sequence alignment further showed that TaBADH-2B terminated at residue 311 and lacked the C-terminal regions corresponding to motifs 7–10 (Figure S1). Among the six intra-wheat homologous pairs, Ka/Ks ratios ranged from 0.033 to 0.085, whereas the two pairs involving *TaBADH-2B* had ratios of 0.050–0.054 (Table S6). All ratios were below 1, consistent with purifying selection acting on the retained aligned codons; however, these estimates do not establish the functional status of *TaBADH-2B*.

### 2.4. Cis-Acting Elements and Synteny Analysis of the TaBADH Family

**Figure 2:**
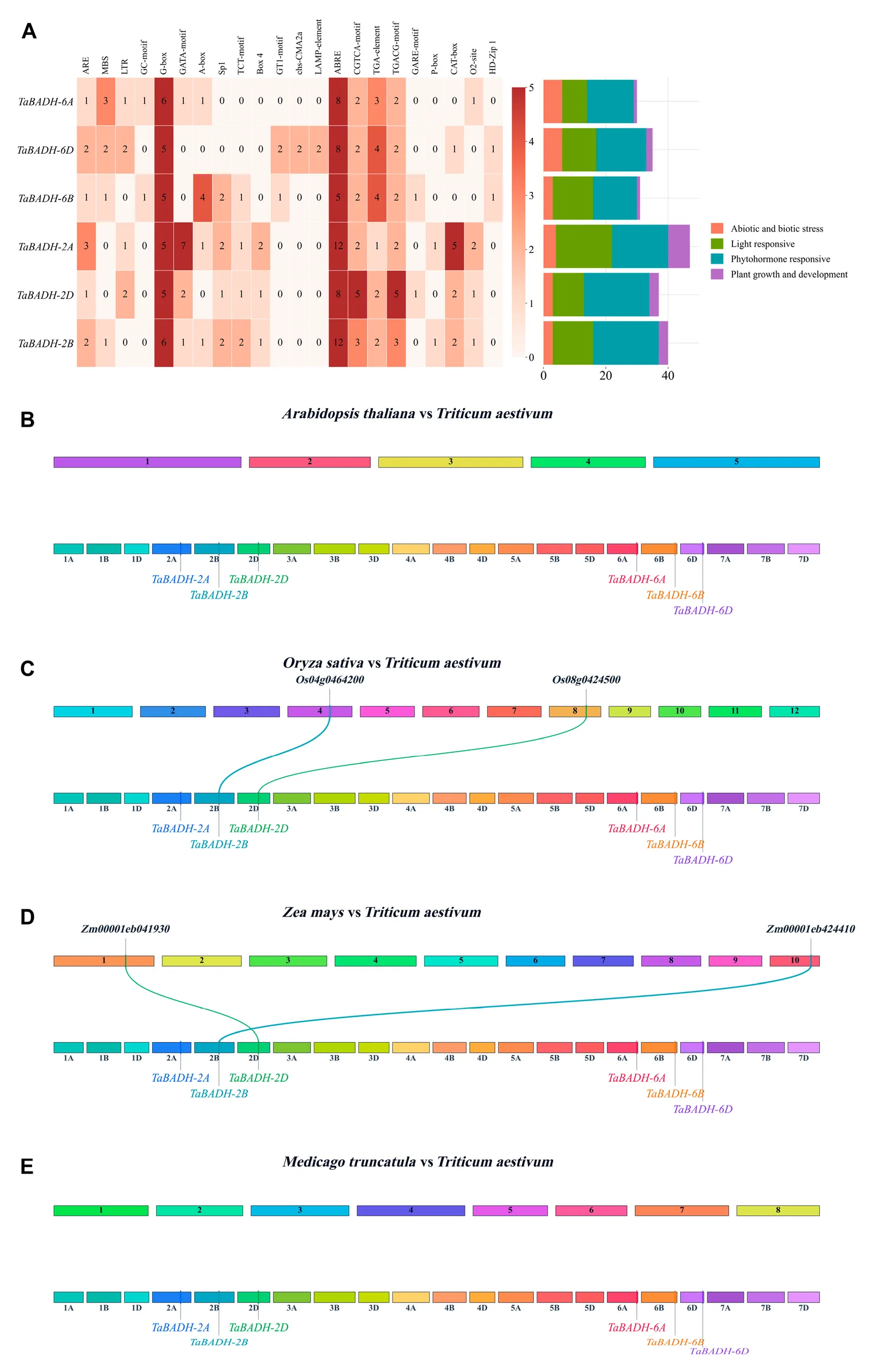
Promoter cis-acting elements and synteny relationships of *TaBADH* genes. (**A**) Distribution of cis-acting elements in the 2000-bp promoter regions of the six *TaBADH* genes. (**B**) *Arabidopsis thaliana* vs. *Triticum aestivum*. (**C**) *Oryza sativa* vs. *Triticum aestivum*. (**D**) *Zea mays* vs. *Triticum aestivum*. (**E**) *Medicago truncatula* vs. *Triticum aestivum*.

A 2000-bp upstream region of each *TaBADH* gene was analyzed using PlantCARE, and 22 classes of cis-acting elements were identified among the six promoters (Figure 2A). These elements were assigned to four functional categories: phytohormone-responsive, light-responsive, abiotic and biotic stress-responsive, and plant growth and development-related elements. Phytohormone-responsive elements constituted the most abundant category and included ABRE, CGTCA-motif, TGACG-motif, GARE-motif, P-box, and TGA-element. Among them, ABRE was the most frequent individual element, occurring 12 times in the *TaBADH-2A* and *TaBADH-2B* promoters, eight times in the *TaBADH-6A*, *TaBADH-6D*, and *TaBADH-2D* promoters, and five times in the *TaBADH-6B* promoter. G-box was detected five to six times in each promoter, and ARE was present in all six promoters with one to three occurrences per promoter. Other stress-related elements, including MBS and LTR, and growth- or development-related elements, including CAT-box, GATA-motif, and O2-site, showed variable distributions among the six promoters. Comparison of selected cis-acting elements showed that the promoters of chromosome group 2 and group 6 *TaBADH* genes shared several elements, including ABRE, G-box, and ARE, but differed in their element combinations. The group 2 promoters generally contained more ABRE motifs and included several development-related elements, such as CAT-box and GATA-motif. All three group 6 promoters contained the ABRE–ARE–G-box–MBS combination. Among the group 2 promoters, this combination was detected only in *TaBADH-2B*, whereas *TaBADH-2A* and *TaBADH-2D* lacked MBS.

Within the wheat genome, collinear relationships were detected between *TaBADH-2A*, *TaBADH-2B*, and *TaBADH-2D* and between *TaBADH-6A*, *TaBADH-6B*, and *TaBADH-6D* (Figure S2). An additional link was observed between *TaBADH-6D* and *TaBADH-2A* (Figure S2). Interspecific synteny analysis identified two collinear *BADH* gene pairs between wheat and rice and two between wheat and maize; no collinear *BADH* pairs were detected between wheat and *Arabidopsis thaliana* or *Medicago truncatula* (Figure 2B–E). Among the four collinear *BADH* gene pairs identified between wheat and rice or maize, Ka/Ks ratios ranged from 0.134 to 0.226 (Table S6) and were all below 1. Ks values exceeded 1 in two of the four gene pairs. Ka/Ks analysis was not performed for the wheat–*Arabidopsis* or wheat–*Medicago* comparisons because no collinear *BADH* gene pairs were identified in these comparisons.

### 2.5. Transcriptomic Responses of Contrasting Wheat Genotypes to Salt Stress

A total of 187.69 Gb of clean data were generated from the 18 RNA-seq libraries, with each sample yielding ≥ 9.90 Gb of clean data and Q30 base percentages exceeding 93.60%. Pairwise correlation analysis indicated high correlations among the biological replicates within each experimental condition (Figure S3). In the PCA, PC1 and PC2 accounted for 19.66% and 15.40% of the total variation, respectively. The samples occupied different positions in the PC1–PC2 space according to genotype and treatment time point, while biological replicates from the same experimental condition were generally located in proximity (Figure S4).

Salt treatment elicited distinct transcriptomic responses in the two genotypes (Figure S5). In Chinese Spring, 3636 DEGs were identified after 24 h of NaCl treatment, including 1258 up-regulated and 2378 down-regulated genes. In Jimai 60, the numbers of DEGs at 8, 24 and 48 h were 3671, 3915 and 3828, respectively. Up-regulated genes outnumbered down-regulated genes at 8 h (2192 vs. 1479) and 24 h (2439 vs. 1476). At 48 h, down-regulated genes outnumbered up-regulated genes (2054 vs. 1774). A direct comparison of the two genotypes identified 5633 DEGs under control conditions, and this number increased to 7131 after 24 h of NaCl treatment (3431 up-regulated and 3700 down-regulated).

### 2.6. GO and KEGG Enrichment of Salt-Responsive DEGs

**Figure 3:**
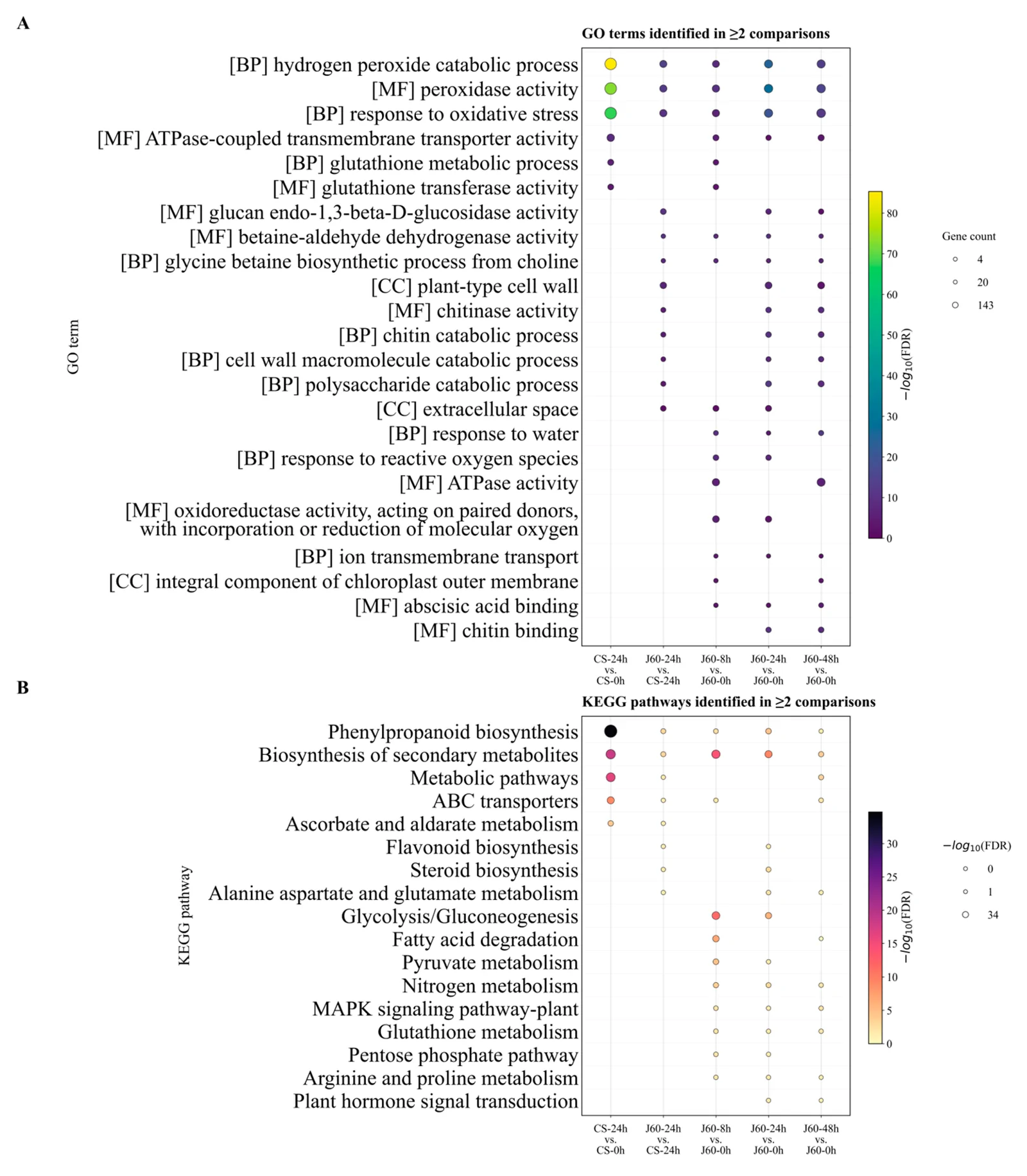
Shared functional enrichment patterns of up-regulated differentially expressed genes across pairwise comparisons. (**A**) Gene Ontology (GO) terms identified in at least two of the five pairwise comparisons. Columns represent CS-24h vs. CS-0h, J60-24h vs. CS-24h, J60-8h vs. J60-0h, J60-24h vs. J60-0h, and J60-48h vs. J60-0h, whereas rows represent individual GO terms. Bubble color represents −log_10_(FDR), and bubble size represents the number of genes assigned to each GO term. (**B**) Kyoto Encyclopedia of Genes and Genomes (KEGG) pathways identified in at least two comparisons. Bubble color and size represent −log_10_(FDR). Blank cells indicate that the corresponding term or pathway was not retained in the original enrichment results for that comparison. The complete GO and KEGG enrichment results for the individual comparisons are provided in Figures S6 and S7, respectively.

GO and KEGG enrichment analysis were performed on the up-regulated salt-responsive DEGs identified in each comparison (Figure 3, Figures S6 and S7, Tables S2 and S3). For clarity, Figure 3 presents selected representative GO terms and KEGG pathways that were identified in at least two comparisons, whereas the complete GO and KEGG enrichment results for the individual comparisons are provided in Figures S6 and S7, respectively. In Chinese Spring, DEGs identified after 24 h of NaCl treatment relative to 0 h (CS-24h vs. CS-0h) were enriched in hydrogen peroxide catabolic process, antioxidant activity, transmembrane transport, extracellular region/apoplast, phenylpropanoid biosynthesis, cutin/suberine/wax biosynthesis and ABC transporters. In Jimai 60, DEGs at 8 h after NaCl treatment (J60-8h vs. J60-0h) were enriched in glycine betaine biosynthetic process from choline, ATPase-coupled transmembrane transport, glycolysis/gluconeogenesis, pyruvate metabolism, fatty acid degradation, MAPK signaling pathway–plant and glutathione metabolism. At 24 h after NaCl treatment (J60-24h vs. J60-0h), enriched terms included glycine betaine biosynthetic process from choline, phenylpropanoid biosynthesis, starch and sucrose metabolism, plant hormone signal transduction, protein processing in endoplasmic reticulum and cell-wall macromolecule catabolic processes. At 48 h after NaCl treatment (J60-48h vs. J60-0h), enriched terms included glycine betaine biosynthetic process from choline, glutathione metabolism, cysteine and methionine metabolism, spliceosome, nucleocytoplasmic transport and ABC transporters. Given the role of BADH in glycine betaine biosynthesis, particular emphasis was placed on enrichment related to glycine betaine metabolism and betaine-aldehyde dehydrogenase activity. The GO term glycine betaine biosynthetic process from choline was identified as enriched in all three Jimai 60 salt-treatment comparisons, namely J60-8h vs. J60-0h, J60-24h vs. J60-0h and J60-48h vs. J60-0h, whereas it was not identified as enriched in the Chinese Spring CS-24h vs. CS-0h comparison. In the cross-genotype comparison at 24 h after NaCl treatment (J60-24h vs. CS-24h), enriched GO terms included glycine betaine biosynthetic process from choline and betaine-aldehyde dehydrogenase activity.

### 2.7. Expression Patterns of TaBADH Genes and qRT-PCR Analysis

**Figure 4:**
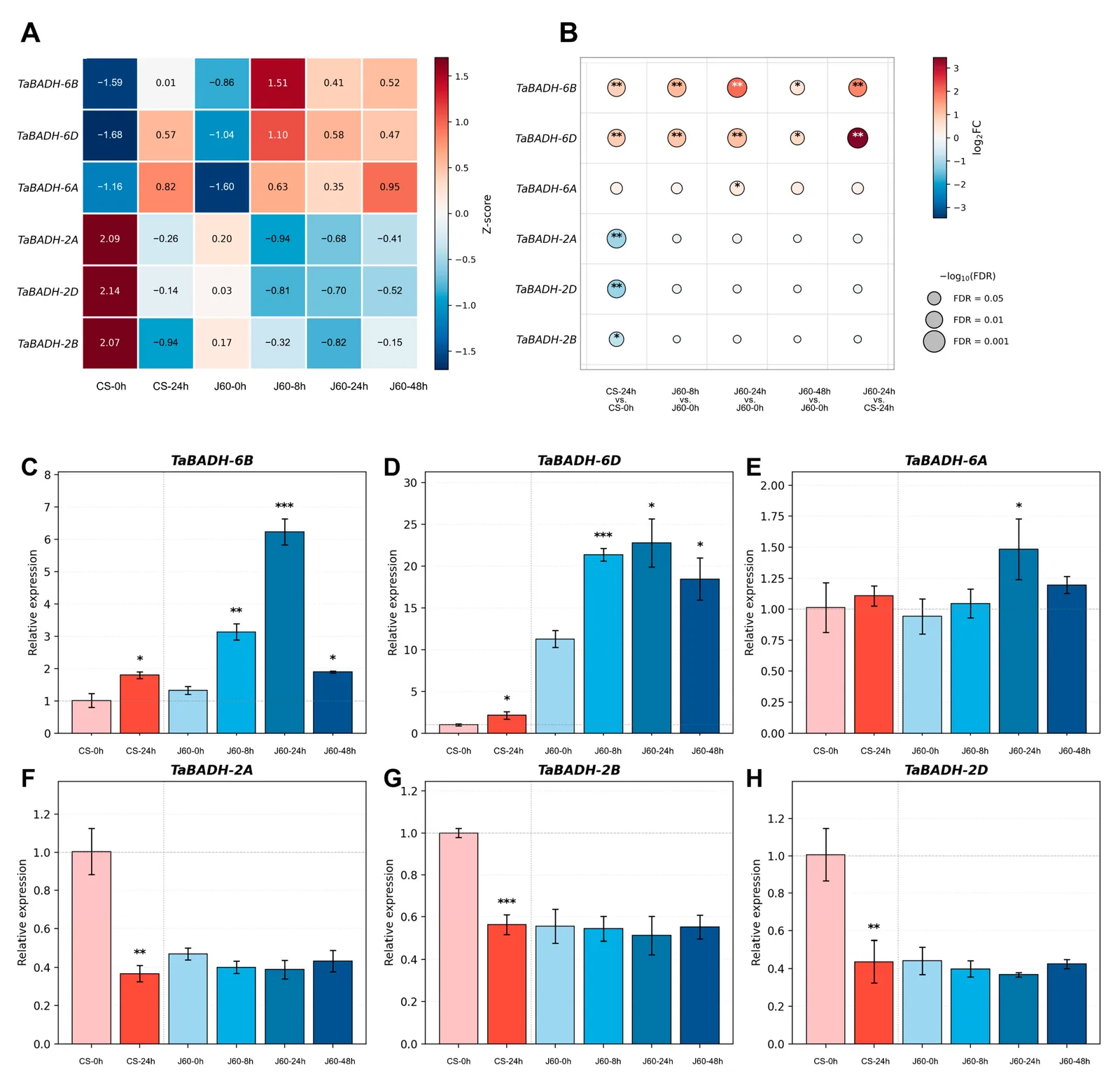
Expression patterns of *TaBADH* genes under 200 mM NaCl treatment and qRT-PCR analysis. (**A**) Heatmap of Z scores calculated from FPKM values for the six *TaBADH* genes across the six experimental conditions. Red and blue represent higher and lower standardized expression levels, respectively. (**B**) Bubble plot of log_2_ fold changes for CS-24h relative to CS-0h; J60-8h, J60-24h, and J60-48h relative to J60-0h; and J60-24h relative to CS-24h. Bubble color denotes the log_2_ fold change, with red and blue indicating positive and negative values, respectively, and bubble size denotes −log_10_(FDR). Asterisks within the bubbles indicate statistical significance (* FDR < 0.05; ** FDR < 0.01). (**C**–**H**) Relative transcript levels of *TaBADH-6B*, *TaBADH-6D*, *TaBADH-6A*, *TaBADH-2A*, *TaBADH-2B*, and *TaBADH-2D*, respectively, determined by qRT-PCR. For each gene, CS-0h was used as the calibrator and assigned a relative expression value of 1. Data are presented as the mean ± SD of three biological replicates. Asterisks indicate significant differences between CS-24h and CS-0h or between each J60 salt-treatment time point and J60-0h, as determined using a two-tailed Welch’s *t*-test in R (* *p* < 0.05; ** *p* < 0.01; *** *p* < 0.001). CS, Chinese Spring; J60, Jimai 60; h, hours after the onset of NaCl treatment.

Expression profiles of the six *TaBADH* genes were extracted from the RNA-seq data (Figure 4A). In Jimai 60, *TaBADH-6B* and *TaBADH-6D* were induced at 8 h of NaCl treatment and remained at elevated levels at 24 h and 48 h. *TaBADH-6A* showed a more modest increase that peaked at 24 h. In contrast, *TaBADH-2A*, *TaBADH-2B* and *TaBADH-2D* showed their highest expression levels in Chinese Spring under control conditions and decreased in all other samples. Differential expression analysis across the five pairwise comparisons revealed distinct patterns between chromosome group 6 and group 2 *TaBADH* members (Figure 4B). *TaBADH-6B* and *TaBADH-6D* were significantly up-regulated (FDR < 0.05) in all five comparisons, with the highest log_2_ fold-change for *TaBADH-6D* observed in J60-24h vs. CS-24h. *TaBADH-6A* was significantly up-regulated only in J60-24h vs. J60-0h. *TaBADH-2A*, *TaBADH-2B* and *TaBADH-2D* were significantly down-regulated in CS-24h vs. CS-0h but did not reach significance in any comparison with Jimai 60 or in J60-24h vs. CS-24h. Among the six *TaBADH* homeologs, *TaBADH-6D* showed the strongest salt-responsive expression within Jimai 60 and was therefore selected for a preliminary heterologous expression assay.

The expression profiles of the six *TaBADH* genes were further examined by qRT-PCR using the same RNA samples as those used for RNA-seq analysis (Figure 4C–H). In Jimai 60, *TaBADH-6D* expression increased at 8 h of NaCl treatment, peaked at 24 h and remained elevated at 48 h. *TaBADH-6B* was up-regulated from 8 h onward and peaked at 24 h. *TaBADH-6A* showed a modest increase at 24 h. *TaBADH-2A*, *TaBADH-2B* and *TaBADH-2D* were significantly down-regulated in Chinese Spring at 24 h relative to CS-0h but showed no significant changes in Jimai 60 at any time point. The expression trends observed by qRT-PCR were consistent with those obtained from the RNA-seq data.

### 2.8. Physiological Responses of Chinese Spring and Jimai 60 Under NaCl Treatment

**Figure 5:**
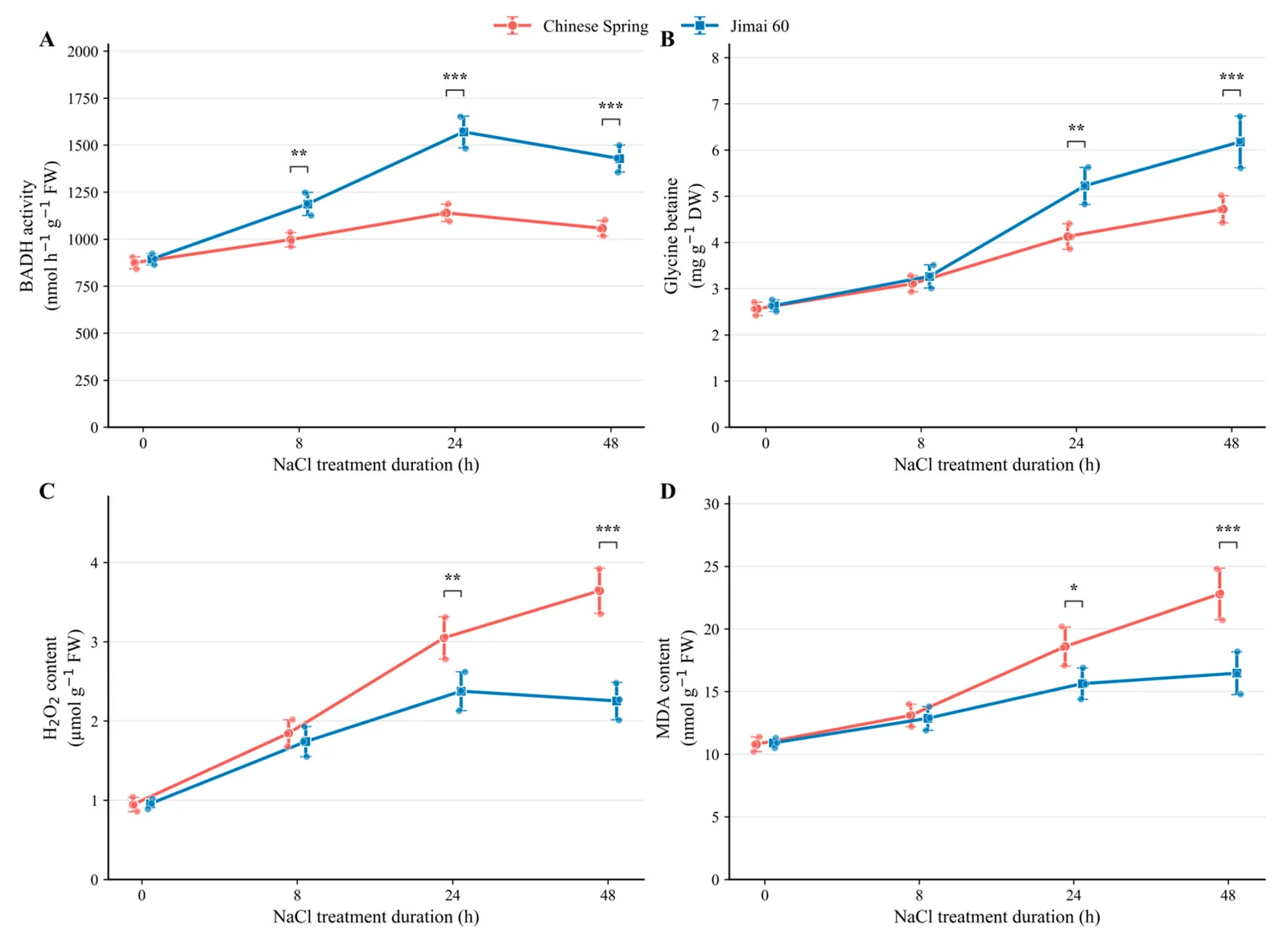
Physiological responses of Chinese Spring and Jimai 60 under 200 mM NaCl treatment. (**A**) BADH activity. (**B**) Glycine betaine content. (**C**) H_2_O_2_ content. (**D**) MDA content. Each point represents one independent biological replicate comprising three pooled whole seedlings from one culture box. Data are presented as means ± SD (*n* = 3). Asterisks indicate significant differences between the two cultivars at the same time point, as determined by two-way ANOVA followed by Holm-adjusted pairwise comparisons (* *p* < 0.05; ** *p* < 0.01; *** *p* < 0.001).

BADH activity and glycine betaine content increased in both cultivars during NaCl treatment, with larger increases in Jimai 60 (Figure 5A,B). In Jimai 60, BADH activity increased from 894.00 ± 29.51 nmol h^−1^ g^−1^ FW at 0 h to 1570.33 ± 84.76 at 24 h and remained at 1428.67 ± 71.53 at 48 h. Glycine betaine content reached 5.23 ± 0.40 mg g^−1^ DW at 24 h and 6.18 ± 0.56 at 48 h in Jimai 60, compared with 4.13 ± 0.28 and 4.72 ± 0.30 in Chinese Spring, respectively. Jimai 60 had significantly higher BADH activity at 8, 24 and 48 h and higher glycine betaine content at 24 and 48 h (Holm-adjusted *p* < 0.05).

H_2_O_2_ and MDA contents also increased in both cultivars, but these increases were smaller in Jimai 60 at 24 and 48 h of NaCl treatment (Figure 5C,D). At 48 h, H_2_O_2_ content was 2.25 ± 0.24 µmol g^−1^ FW in Jimai 60 and 3.64 ± 0.29 in Chinese Spring, whereas MDA content was 16.47 ± 1.70 and 22.80 ± 2.05 nmol g^−1^ FW, respectively. Jimai 60 had significantly lower H_2_O_2_ and MDA contents at 24 and 48 h (Holm-adjusted *p* < 0.05). Two-way ANOVA detected significant genotype × treatment-duration interactions for all four traits (*p* < 0.01).

### 2.9. Functional Enrichment and Predicted Protein–Protein Interaction Network of Salt-Responsive DEGs

**Figure 6:**
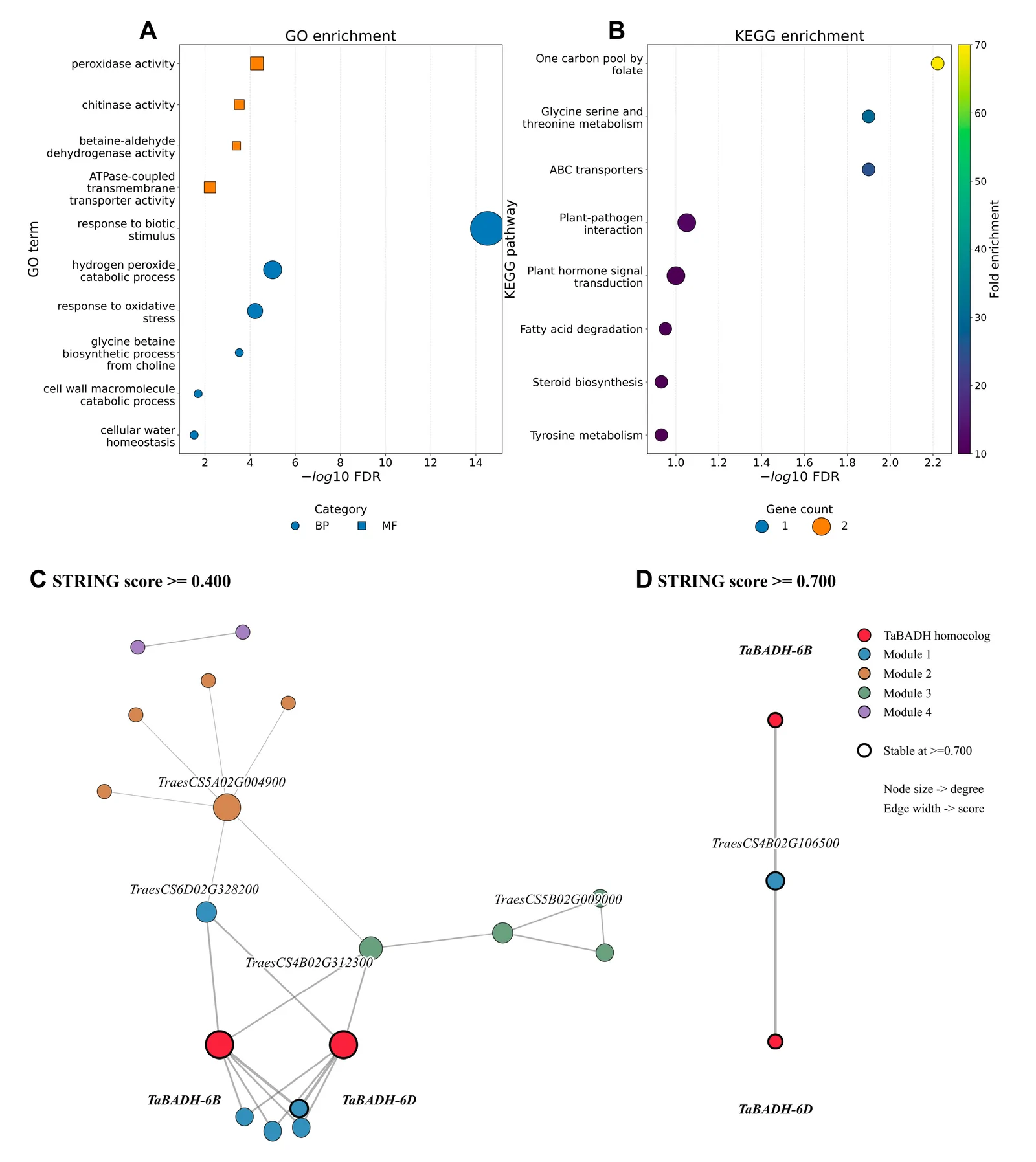
Functional enrichment and predicted STRING interaction network of the intersection DEG set. (**A**) GO enrichment results; the x-axis shows −log_10_(FDR), and point shape and color distinguish the GO category (circles, biological process, BP; squares, molecular function, MF). (**B**) KEGG pathway enrichment results; the x-axis shows −log_10_(FDR), bubble color indicates fold enrichment, and bubble size represents gene count. (**C**) Predicted network generated with a STRING confidence score threshold of 0.400. (**D**) Predicted network generated with a STRING confidence score threshold of 0.700.

To identify genes potentially associated with the salt-tolerant phenotype of Jimai 60, DEGs shared by the three within-genotype comparisons in Jimai 60 (J60-8h vs. J60-0h, J60-24h vs. J60-0h and J60-48h vs. J60-0h) were first intersected, and the resulting set was then intersected with the DEGs from the cross-genotype comparison J60-24h vs. CS-24h. The final intersection set comprised genes that responded consistently to NaCl treatment in Jimai 60 across all three time points and were also differentially expressed between Jimai 60 and Chinese Spring at 24 h. GO enrichment analysis of the intersection set (Figure 6A, Table S4) identified glycine betaine biosynthetic process from choline, betaine-aldehyde dehydrogenase activity, hydrogen peroxide catabolic process, peroxidase activity, response to oxidative stress and response to biotic stimulus. KEGG enrichment analysis (Figure 6B, Table S5) identified one carbon pool by folate, glycine, serine and threonine metabolism, and ABC transporters as the three pathways with the lowest FDR values. The predicted STRING network constructed from the intersection set was analyzed at two confidence thresholds (Figure 6C,D). At a confidence score of ≥0.400, *TaBADH-6B* and *TaBADH-6D* were connected with multiple DEGs and had the highest node degrees in the network. At a confidence score of ≥0.700, only the predicted edges linking *TaBADH-6B* and *TaBADH-6D* with *TraesCS4B02G106500* were retained.

### 2.10. Heterologous Expression of TaBADH-6D in E. coli Under NaCl Treatment

**Figure 7:**
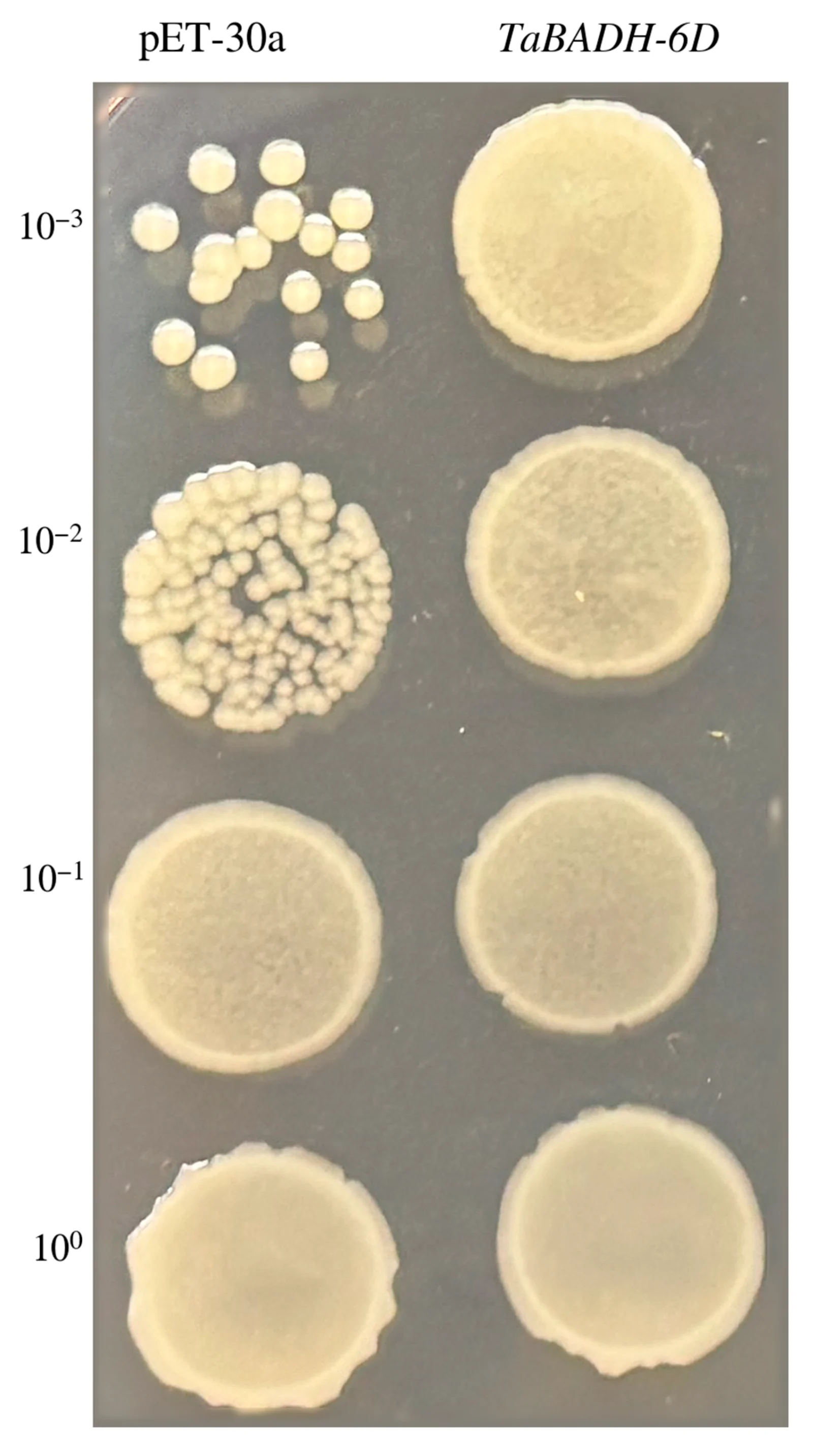
Salt tolerance assay of *E. coli* cells expressing *TaBADH-6D*. Growth of *E. coli* BL21 (DE3) cells carrying pET-30a-*TaBADH-6D* or the empty pET-30a vector on LB plates containing 200 mM NaCl. Cells carrying the empty vector served as the control.

After IPTG induction, *E. coli* BL21 (DE3) strains harboring pET-30a-*TaBADH-6D* or the empty pET-30a vector were normalized to the same OD_600_, serially diluted (10^0^–10^−3^) and spotted onto LB plates containing 200 mM NaCl (Figure 7). Growth of the empty vector strain was suppressed under 200 mM NaCl, whereas the *TaBADH-6D*-expressing strain produced larger and denser colonies than the control at each dilution. These results indicate that *TaBADH-6D* expression was associated with improved *E. coli* growth under this condition.

## 3. Discussion

Although bread wheat has a large allohexaploid genome, only six *TaBADH* genes were identified here, consistent with observations in other monocots that *BADH* gene number does not simply scale with genome size or ploidy level [23,27,28]. This pattern suggests that *BADH* copy number in wheat may reflect gene retention and loss during genome evolution, rather than genome expansion alone [29,30]. In wheat, *TaBADH*s were located on group 2 and group 6 chromosomes. Collinear links were detected among homeologous chromosomes, and syntenic relationships were observed with rice and maize homologs, supporting a conserved monocot-related background for most *TaBADH* members rather than recent large-scale expansion [23,27,28]. The four wheat–rice or wheat–maize collinear *BADH* pairs had Ka/Ks values below 1, consistent with a predominant signal of purifying selection in their retained aligned coding regions. No corresponding evolutionary-rate comparison was made for *Arabidopsis* or *Medicago* because no clear collinear *BADH* pairs were detected. Thus, the absence of detectable collinearity is compatible with deep monocot–dicot divergence [31,32]. Notably, five TaBADH proteins were 502–506 aa in length, whereas TaBADH-2B was 311 aa long and lacked the C-terminal region corresponding to motifs 7–10. The C-terminal multiple sequence alignment confirmed this structural difference. Pairwise Ka/Ks values for *TaBADH-2B* versus *TaBADH-2A* or *TaBADH-2D* were below 1, but these estimates covered only the retained alignable codons. Therefore, the current evidence supports clear structural divergence of *TaBADH-2B* without establishing pseudogenization, catalytic inactivity or functional degeneration.

The shared presence of ABRE, G-box and ARE motifs indicates that the six *TaBADH* promoters contain common hormone-, light- and stress-related cis-regulatory elements. However, the number of ABRE motifs did not match the magnitude of the transcriptional response of *TaBADH* genes under salt stress. ABRE motifs provide potential binding sites for AREB/ABF transcription factors in ABA-dependent signaling [33], and ABA signaling and ABF/AREB genes have been reported to participate in wheat responses to salt stress, including under 200 mM NaCl treatment [34,35]. In this study, *TaBADH-2A* and *TaBADH-2B* contained the highest numbers of ABRE motifs, but their transcript levels did not increase in Jimai 60 after NaCl treatment. In contrast, *TaBADH-6D* showed the strongest salt-induced expression, although its promoter did not contain the highest number of ABRE motifs. This mismatch suggests that ABRE abundance alone does not determine the salt-responsive expression of *TaBADH* genes.

ABRE function depends on promoter context rather than copy number alone. ABA responsive transcription can require the coordinated action of ABREs with nearby coupling elements [36,37], whereas chromatin accessibility and chromatin modifications can influence the access of transcription factors to cis-regulatory sites [38,39]. Thus, the greater ABRE abundance in the *TaBADH-2A* and *TaBADH-2B* promoters would not necessarily result in stronger transcriptional induction if these sites were inaccessible or occurred in less favorable regulatory contexts. Differences in cis-acting element combinations may also contribute to the contrasting expression patterns. All three group 6 promoters contained the ABRE–ARE–G-box–MBS combination. Among the group 2 promoters, this combination was present only in *TaBADH-2B*, whereas *TaBADH-2A* and *TaBADH-2D* lacked MBS. However, because this element combination occurred in genes with different expression profiles, its presence alone cannot explain the strong induction of *TaBADH-6D.* Instead, local promoter context, chromatin accessibility, and transcription-factor occupancy may collectively contribute to the observed expression differences.

Although the two cultivars produced similar numbers of DEGs, their transcriptional responses differed in direction. At the shared 24 h time point, down-regulated genes predominated in Chinese Spring, whereas up-regulated genes predominated in Jimai 60. The prevalence of transcriptional induction in Jimai 60 is consistent with observations in other salt-tolerant wheat genotypes [40,41,42]. Functional enrichment further showed that the salt-responsive DEG sets involved processes related to ROS detoxification, phenylpropanoid and glutathione metabolism, membrane transport, and glycine betaine biosynthesis from choline [40,41,43]. These processes contribute to redox regulation, osmotic adjustment, and solute transport during plant responses to salinity. Thus, the contrasting directions of transcriptomic change may reflect differences in the extent to which the two cultivars mobilized protective responses under NaCl treatment. Notably, the GO term glycine betaine biosynthetic process from choline was identified as enriched in all three Jimai 60 salt-treatment comparisons, whereas this term was not enriched in Chinese Spring at 24 h. In the cross-genotype comparison under salt stress, genes expressed at higher levels in Jimai 60 were also enriched for betaine-aldehyde dehydrogenase activity. These enrichment patterns highlighted a *BADH*-related glycine betaine biosynthetic response in Jimai 60 within the broader transcriptomic response. This enrichment pattern was consistent with the expression profiles of *TaBADH* genes. Under salt stress, the six *TaBADH* genes displayed two contrasting expression patterns. The group 6 members, especially *TaBADH-6D* and *TaBADH-6B*, were up-regulated in Jimai 60 at the sampled time points. The group 2 members were not induced in Jimai 60 and were down-regulated in Chinese Spring at 24 h. These patterns indicate that group 6 members accounted for most of the salt-responsive *TaBADH* expression detected in Jimai 60, with *TaBADH-6D* showing the strongest response. Homeolog-biased responses have been reported for several wheat genes involved in salt tolerance. For example, *TaSOS1-D* is induced by salt stress in roots and contributes more strongly to salt tolerance than the other *TaSOS1* homeologs in transgenic *Arabidopsis* [26]. Natural variation in *TaSPL6-D* improves salt tolerance by regulating *TaHKT1;5-D* expression and Na^+^ exclusion while maintaining yield-related traits [44]. These studies demonstrate that individual homeologs can contribute more strongly than their counterparts in salt-related traits in wheat.

The physiological measurements were consistent with the cultivar-level transcriptional pattern. Compared with Chinese Spring, Jimai 60 showed higher *BADH* activity and glycine betaine content and lower H_2_O_2_ and MDA contents at 24 and 48 h. At 24 h, the coordinated downregulation of *TaBADH-2A*, *TaBADH-2B*, and *TaBADH-2D* in Chinese Spring occurred across the A, B, and D subgenomes and coincided with lower *BADH* activity and glycine betaine content and higher H_2_O_2_ and MDA contents than those in Jimai 60. This association suggests that the reduced expression of chromosome group 2 homeologs may be related to lower glycine betaine accumulation and greater oxidative damage in Chinese Spring. Thus, greater glycine betaine accumulation was accompanied by lower H_2_O_2_ and MDA contents during prolonged NaCl treatment. This pattern was consistent with the enrichment of the glycine betaine biosynthetic process and the sustained expression of group 6 *TaBADH* genes in Jimai 60. However, total BADH activity reflects the combined activity of BADH proteins and does not resolve the contribution of a single homeolog.

Among the six *TaBADH* homeologs, *TaBADH-6D* showed the most consistent salt-induced response. In the predicted STRING network, *TaBADH-6D* showed a high node degree, and its edge with *TraesCS4B02G106500* was retained at a confidence score of ≥0.700. *TraesCS4B02G106500* has been identified as *TaADH10*, a predicted cytoplasmic alcohol dehydrogenase [45]. The retention of its association with *TaBADH-6D* at a STRING score of ≥0.700 places these two oxidoreductases within the same predicted stress responsive network and may reflect coordination between glycine betaine metabolism and cellular redox metabolism under NaCl treatment. However, this predicted association does not establish a direct physical interaction or regulatory direction and requires experimental validation. The qRT-PCR analysis showed an expression pattern consistent with the RNA-seq data, with *TaBADH-6D* transcript levels increasing at 8 h and remaining elevated at 24 and 48 h in Jimai 60. However, because the qRT-PCR assays were performed using the same RNA samples as those used for RNA-seq, these results support the RNA-seq expression trends through an alternative analytical approach but do not demonstrate their reproducibility in an independent biological experiment. Further experiments using independently grown plants and separately collected tissue samples are needed to confirm the salt-responsive expression profiles of the *TaBADH* genes. The *E. coli* strain expressing *TaBADH-6D* showed better growth than the empty vector control under 200 mM NaCl. This result suggests that the expression of *TaBADH-6D* was associated with improved bacterial growth under the tested condition. However, certain protein modifications occurring in plant cells may not occur in *E. coli*, which could affect the stability or activity of the expressed protein. Therefore, the observed bacterial phenotype provides preliminary evidence from a heterologous system but does not directly establish the function of *TaBADH-6D* in wheat. Plant-level overexpression or genome-editing studies will therefore be required to establish the contribution of *TaBADH-6D* to salt tolerance in wheat [46,47].

## 4. Materials and Methods

### 4.1. Plant Materials and Salt-Stress Treatment

Seeds of Jimai 60 and Chinese Spring were provided by the School of Life Science and Engineering, Jining University. Previous evaluations documented contrasting salt responses in Jimai 60 and Chinese Spring [48,49]. Jimai 60 was further identified as salt tolerant at the germination and seedling stages [49], whereas Chinese Spring was characterized as salt sensitive based on its physiological and transcriptomic responses to NaCl stress [50]. Jimai 60 has also been evaluated under coastal saline field conditions over two consecutive growing seasons [51]. Plump and uniformly sized seeds were surface-sterilized in 1.5% (*w*/*v*) sodium hypochlorite for 10 min, rinsed two to three times with distilled water, and blotted dry on sterile filter paper. The sterilized seeds were then imbibed at 4 °C for 48 h to synchronize germination. Uniformly germinated seeds were transferred to plastic germination boxes lined with two layers of filter paper, with 40 seeds per box. Salt treatment was initiated immediately after transfer, and the boxes were then placed in the growth chamber; thus, the experiment assessed the seedling-stage response to salt stress. A concentration of 200 mM NaCl was selected based on previous studies of wheat seedlings, in which this concentration induced clear physiological and transcriptional responses to salt stress [34,40]. The boxes were covered with lids to minimize evaporation and incubated in a growth chamber at 25 °C under a 14 h light/10 h dark photoperiod and 60% relative humidity. For Jimai 60, whole seedlings were collected at 0, 8, 24 and 48 h after the onset of NaCl treatment; for Chinese Spring, whole seedlings were collected at 0 and 24 h. Three whole seedlings were pooled to form one biological replicate, with three biological replicates per time point. Samples were immediately frozen in liquid nitrogen and stored at −80 °C until RNA extraction.

### 4.2. Genome-Wide Identification of BADH Family Members in Wheat

The wheat genome sequence, protein sequences and genome annotation files were retrieved from the Ensembl Plants database (https://plants.ensembl.org/) [52]. BADH protein sequences from rice (*Oryza sativa*), maize (*Zea mays*), *Arabidopsis thaliana* and *Medicago truncatula* were downloaded from the UniProt database (https://www.uniprot.org/) [53] and used as reference sequences. The selected reference sequences were aligned and used to construct a profile hidden Markov model with HMMER 3.0 [54]. The resulting model was searched against the complete wheat protein dataset using an E-value threshold of ≤1 × 10^−5^ and a domain score threshold of ≥20. The E-value threshold was used to exclude weak sequence matches, whereas the domain score threshold was applied to retain candidates with sufficiently strong similarity across the matched BADH region. Candidate proteins meeting both HMM search criteria were subsequently examined using InterPro (https://www.ebi.ac.uk/interpro/) [55]. A sequence was retained as a TaBADH candidate only when the characteristic ALDH/BADH domain was confirmed, whereas sequences lacking this domain were excluded.

### 4.3. Physicochemical Characterization of TaBADH Proteins

The physicochemical properties of the TaBADH proteins, including the number of amino acids, molecular weight, theoretical isoelectric point (pI), instability index, aliphatic index and grand average of hydropathicity (GRAVY), were predicted using the ExPASy ProtParam online tool (https://web.expasy.org/protparam/, accessed on 23 August 2026) [56]. Subcellular localization of the TaBADH proteins was predicted using Plant-mPLoc (http://www.csbio.sjtu.edu.cn/bioinf/plant-multi/, accessed on 23 August 2026) [57].

### 4.4. Phylogenetic Analysis and Chromosomal Localization of TaBADH Genes

Multiple sequence alignment was performed for the TaBADH proteins and BADH proteins from rice, maize, *Arabidopsis thaliana*, *Medicago sativa* and four representative Triticeae species, namely *Triticum urartu*, *Aegilops tauschii*, *Hordeum vulgare* and *Secale cereale*, using MUSCLE 3.8.31 [58]. Poorly aligned positions were removed using trimAl 1.4 [59]. A maximum-likelihood tree was constructed using IQ-TREE 3.1.2 [60] with the LG+G4 model and 1000 ultrafast bootstrap replicates. The tree was rooted with dicot BADH proteins, and bootstrap values ≥ 70% were displayed at the corresponding nodes. The resulting tree was visualized and annotated using the Biomuse online platform (https://www.biomuse.cn/, accessed on 23 August 2026). The chromosomal positions of the *TaBADH* genes were extracted from the wheat genome annotation file and visualized using the Biomuse online platform (https://www.biomuse.cn/, accessed on 23 August 2026).

### 4.5. Conserved Motif and Gene Structure Analysis of TaBADH

Conserved motifs in the TaBADH proteins were identified using the MEME online tool (https://meme-suite.org/meme/tools/meme, accessed on 23 August 2026) [61], with the maximum number of motifs set to 10 and other parameters left at their default values. The exon–intron structures of the *TaBADH* genes were extracted from the wheat genome annotation file. Conserved motif distributions and gene structures were then visualized together with the phylogenetic tree using TBtools v2.515. To further assess the C-terminal structural divergence of TaBADH-2B, the six TaBADH protein sequences were aligned with MUSCLE 3.8.31, and the alignment was compared with the MEME motif coordinates.

### 4.6. Cis-Acting Element and Synteny Analysis of TaBADH Genes

The 2000-bp upstream genomic sequence of each *TaBADH* gene was extracted using the Biomuse online platform (https://www.biomuse.cn/, accessed on 23 August 2026) and submitted to PlantCARE (https://bioinformatics.psb.ugent.be/webtools/plantcare/html/, accessed on 23 August 2026) [62] for prediction of cis-acting elements. The predicted elements were classified by functional category and visualized in R v4.3.2. To complement the comparison of total element counts, we extracted the identity, copy number and genomic position of representative core elements associated with hormone response, stress regulation and development, including ABRE, ARE, G-box, MBS, LTR, CAT-box and GATA-motif. These elements were compared across the six *TaBADH* promoters and between chromosome group 2 and group 6 promoters to characterize element combinations and subgroup-specific promoter features. Synteny analysis between wheat and each of rice, maize, *Arabidopsis* and *Medicago truncatula* were performed using the One Step MCScanX module of TBtools v2.515 [63], and the resulting collinear relationships were visualized in R v4.3.2 using the circlize [64] and RIdeogram [65] packages. For evolutionary-rate analysis, protein sequences for six intra-wheat homologous pairs and four wheat–rice or wheat–maize collinear *BADH* pairs were aligned with MUSCLE 3.8.31. Corresponding coding sequences were mapped to the protein alignments, codon columns containing a gap in either sequence were excluded, and Ka and Ks were estimated using the Nei–Gojobori method (NG86) implemented in Biopython 1.87 [66]. Ka/Ks was calculated for the retained aligned codons.

### 4.7. Differential Expression, Functional Enrichment and Protein–Protein Interaction Network Analysis

RNA sequencing was performed by Beijing Biomarker Technologies Co., Ltd. (Beijing, China). The mRNA was enriched using oligo(dT) magnetic beads, fragmented, reverse-transcribed into cDNA, ligated with sequencing adapters and amplified to construct cDNA libraries. The qualified libraries were sequenced using a paired-end 150-bp strategy. After adapter-contaminated and low-quality reads were removed, clean reads were aligned to the IWGSC RefSeq v2.1 wheat reference genome using HISAT2 [67]. Transcript assembly and gene-expression quantification were performed using StringTie [68]. Gene expression was quantified as fragments per kilobase of transcript per million mapped fragments (FPKM); the FPKM values were used to describe and visualize expression patterns. Differential expression analysis was performed using DESeq2 v1.40.2 based on the gene-level count matrix [69]. Each pairwise comparison included three biological replicates per group. Count data were normalized using the sample-specific size-factor normalization implemented in DESeq2 v1.40.2 to account for differences in sequencing depth and library composition. Six pairwise comparisons were performed based on the sample grouping: CS-24h vs. CS-0h for Chinese Spring at 24 h versus 0 h after NaCl treatment; J60-8h vs. J60-0h, J60-24h vs. J60-0h and J60-48h vs. J60-0h for Jimai 60 at 8, 24 and 48 h versus 0 h after NaCl treatment, respectively; J60-0h vs. CS-0h for the cross-genotype comparison under control conditions; and J60-24h vs. CS-24h for the cross-genotype comparison at 24 h after NaCl treatment. Because Chinese Spring was sampled only at 0 h and 24 h, direct genotype comparisons were restricted to the shared 0 h and 24 h time points, whereas the 8 h and 48 h samples were used only to analyze the temporal salt response within Jimai 60. *p* values obtained from differential expression testing were adjusted for multiple comparisons using the Benjamini–Hochberg procedure. Genes with |log_2_ fold change| ≥ 1 and FDR < 0.01 were defined as differentially expressed genes. This threshold applied to all transcriptome-wide analysis; individual *TaBADH* significance (Figure 4B) was annotated directly at FDR < 0.05, <0.01. The DEGs were subsequently subjected to Gene Ontology and Kyoto Encyclopedia of Genes and Genomes enrichment analysis [70,71]. Protein–protein interactions among the encoded products were predicted using the STRING database (https://string-db.org/, accessed on 23 August 2026) [72].

### 4.8. Quantitative Real-Time PCR (qRT-PCR) Analysis

The six *TaBADH* family members were selected for qRT-PCR analysis. *Actin* was used as the internal reference gene. Gene-specific primers (Table S1) were designed using Primer3 Plus (https://www.primer3plus.com/, accessed on 23 August 2026) [73] based on sequence comparison between the six *TaBADH* coding regions. Primer-binding sites containing homeolog-specific nucleotide differences were preferentially selected. Each primer pair was further aligned against all six *TaBADH* coding sequences, and only primer pairs without complete matches to non-target homeologs were retained. Total RNA samples returned from the sequencing service provider were used to prepare cDNA templates. Amplification was performed with the SYBR Premix Pro Taq HS qPCR Kit (Accurate Biology Co., Ltd., Changsha, China) on a Bio-Rad CFX96 real-time PCR system (Bio-Rad, Hercules, CA, USA). Each 10 μL reaction contained 100 ng cDNA, 1 μL of each forward and reverse primer (10 μM), 5 μL of 2× SYBR Premix Pro Taq HS, and nuclease-free water to a final volume of 10 μL. The thermal cycling program consisted of an initial denaturation at 95 °C for 3 min, followed by 40 cycles of 95 °C for 10 s, 58 °C for 10 s and 72 °C for 30 s, and a final melting curve analysis from 65 to 95 °C with 0.5 °C increments. Relative expression levels were calculated using the 2^−ΔΔCt^ method [74], with CS-0h used as the calibrator and assigned a value of 1. Three biological replicates were analyzed for each experimental condition, with three technical replicates performed for each biological replicate. The technical replicates were averaged before calculating the relative expression level of each biological replicate. Statistical comparisons were performed in R v4.3.2 using two-tailed Welch’s *t*-test based on the relative expression values of the three biological replicates. Differences were considered statistically significant at *p* < 0.05.

### 4.9. Determination of BADH Activity, Glycine Betaine, H_2_O_2_ and MDA

For the physiological assays, whole seedlings of Chinese Spring and Jimai 60 were collected at 0, 8, 24 and 48 h after 200 mM NaCl treatment. Three independent biological replicates were analyzed for each genotype and time point. Each replicate comprised three whole seedlings pooled from one independent culture box. *BADH* activity, glycine betaine content, H_2_O_2_ content and MDA content were determined using the G0176W, G0122W, G0168W48 and G0109W48 microplate assay kits, respectively (Suzhou Grace Biotechnology Co., Ltd., Suzhou, China), according to the manufacturer’s instructions. Absorbance was measured using a microplate reader. Fresh tissue was used for BADH activity, H_2_O_2_ and MDA measurements, whereas glycine betaine was determined using dried tissue and expressed on a dry-weight basis. *BADH* activity, glycine betaine, H_2_O_2_ and MDA were expressed as nmol h^−1^ g^−1^ FW, mg g^−1^ DW, µmol g^−1^ FW and nmol g^−1^ FW, respectively. Data were analyzed by two-way ANOVA with genotype and treatment duration as fixed factors. Pairwise comparisons between genotypes at each time point were adjusted using the Holm method, with *p* < 0.05 considered statistically significant.

### 4.10. Prokaryotic Expression of TaBADH-6D and Assessment of Salt Tolerance in E. coli

The complete coding sequence of *TaBADH-6D* was amplified by PCR from cDNA prepared from salt-treated Jimai 60 seedlings using gene-specific primers. Following gel purification and double digestion with BamHI and EcoRI, the PCR product was ligated into the pET-30a vector using T4 DNA ligase at 16 °C overnight to generate pET-30a-*TaBADH-6D*. The ligation product was transformed into *E. coli* DH5α competent cells; individual positive colonies were verified by colony PCR and sequencing, and the recombinant plasmid was isolated and transformed into *E. coli* BL21 (DE3) competent cells. BL21 (DE3) cells transformed with the empty pET-30a vector were used as a control. Single positive colonies were inoculated into liquid LB medium containing 50 mg L^−1^ kanamycin and cultured overnight at 37 °C with shaking at 200 rpm. After 16 h, the cultures were subcultured into fresh LB medium at a 1:100 dilution and grown to an OD_600_ of 0.6–0.8, at which point isopropyl β-D-1-thiogalactopyranoside (IPTG) was added to a final concentration of 1.0 mM and the cells were induced for 3 h. After induction, cells were collected by centrifugation, resuspended in fresh LB medium and adjusted to the same OD600 and serially diluted in liquid LB to 10^0^, 10^−1^, 10^−2^ and 10^−3^. Aliquots (10 μL) of each dilution were spotted onto LB agar plates containing 50 mg L^−1^ kanamycin, 1.0 mM IPTG and 200 mM NaCl. Plates were incubated at 37 °C for 16–24 h, after which colony growth was photographed.

## 5. Conclusions

In this study, we identified six *BADH* genes in bread wheat, located on chromosomes 2A, 2B, 2D, 6A, 6B and 6D. The six genes were classified into two groups based on phylogeny and gene structure: the three group 6 members share similar protein lengths and a complete set of conserved motifs, while TaBADH-2B is shorter than the other five and lacks several of these motifs. Under 200 mM NaCl, group 6 genes were induced in the salt-tolerant cultivar Jimai 60, whereas group 2 genes were not. Jimai 60 also showed higher BADH activity and glycine betaine content and lower H_2_O_2_ and MDA contents at later treatment times. Among the six genes, *TaBADH-6D* showed the strongest induction in both RNA-seq and qRT-PCR, the highest node degree in the STRING network among the six *TaBADH* members, and its heterologous expression was associated with improved *E. coli* growth under 200 mM NaCl. These results support the prioritization of *TaBADH-6D* for further study, but its role in wheat salt tolerance requires direct plant-level validation, for example, through transgenic overexpression or genome editing.

## Figures and Tables

**Table 1:** Physicochemical properties of TaBADH proteins.

Gene ID	Gene Name	Amino Acids (aa)	Molecular Weight (Da)	pI	Instability Index	GRAVY	Aliphatic Index	Predicted Subcellular Location
* TraesCS2A02G336000 *	* TaBADH-2A *	506	54,626.96	5.88	32.54	0.029	94.72	Chloroplast, cytoplasm, peroxisome
* TraesCS2B02G346000 *	* TaBADH-2B *	311	32,598.77	5.06	34.07	0.264	99.94	Chloroplast, peroxisome, cytoplasm
* TraesCS2D02G326900 *	* TaBADH-2D *	506	54,602.95	5.77	31.11	0.028	94.53	Peroxisome, chloroplast, cytoplasm
* TraesCS6A02G371100 *	* TaBADH-6A *	502	54,289.27	5.46	35.34	−0.066	88.69	Chloroplast, cytoplasm, peroxisome
* TraesCS6B02G408100 *	* TaBADH-6B *	503	54,424.40	5.52	35.67	−0.069	88.71	Chloroplast, cytoplasm, peroxisome
* TraesCS6D02G355000 *	* TaBADH-6D *	503	54,426.42	5.52	34.76	−0.074	88.51	Cytoplasm, chloroplast, peroxisome

## Data Availability

The RNA-seq raw data generated in this study are available in the National Genomics Data Center Genome Sequence Archive (https://ngdc.cncb.ac.cn/) under BioProject PRJCA063797.

## Supplementary Materials

The following supporting information can be downloaded at: https://www.mdpi.com/article/10.3390/plants15172577/s1, Figure S1: Structural divergence of TaBADH-2B relative to the other five TaBADH proteins, including protein lengths, conserved motif distributions and a multiple sequence alignment of the C-terminal regions corresponding to motifs 7–10; Figure S2: Intragenomic collinear relationships among the six *TaBADH* loci in wheat; Figure S3: Pairwise Pearson correlation heatmap for the 18 RNA-seq libraries; Figure S4: Principal component analysis of the 18 RNA-seq libraries; Figure S5: Numbers of up- and down-regulated genes in the six pairwise comparisons; Figure S6: Complete GO enrichment results for up-regulated DEGs in five pairwise comparisons; Figure S7: Complete KEGG enrichment results for up-regulated DEGs in five pairwise comparisons; Table S1: Gene-specific primers used for quantitative RT-PCR (qRT-PCR); Table S2: GO enrichment analysis of differentially expressed genes under NaCl treatment; Table S3: KEGG enrichment analysis of differentially expressed genes under NaCl treatment; Table S4: GO enrichment analysis of the intersection DEG set; Table S5: KEGG enrichment analysis of the intersection DEG set; Table S6: Ka, Ks and Ka/Ks values of intra-wheat *TaBADH* homologous pairs and inter-species collinear *BADH* pairs.

## References

[1] Hopmans J.W., Qureshi A.S., Kisekka I., Munns R., Grattan S.R., Rengasamy P., Ben-Gal A., Assouline S., Javaux M., Minhas P.S. (2021). Critical knowledge gaps and research priorities in global soil salinity. Adv. Agron..

[2] Food and Agriculture Organization of the United Nations (2024). Global Status of Salt-Affected Soils: Main Report.

[3] Wang G., Ni G., Feng G., Burrill H.M., Li J., Zhang J., Zhang F. (2024). Saline-alkali soil reclamation and utilization in China: Progress and prospects. Front. Agric. Sci. Eng..

[4] Ehtaiwesh A., Sunoj V.S.J., Djanaguiraman M., Prasad P.V.V. (2024). Response of winter wheat genotypes to salinity stress under controlled environments. Front. Plant Sci..

[5] Shabala S., Chen X., Yun P., Zhou M. (2026). Salinity tolerance in wheat: Rethinking the targets. J. Exp. Bot..

[6] Zhao L., Li S., He X., Liu H., Cheng Y., Wang Y., Kang H., Zeng J. (2025). Identification of salt-tolerant cultivars and plant traits in wheat during germination and seedling emergence stages. Plant Soil Environ..

[7] Javid S., Bihamta M.R., Omidi M., Abbasi A.R., Alipour H., Ingvarsson P.K., Poczai P. (2025). Genome-wide association study (GWAS) uncovers candidate genes linked to the germination performance of bread wheat (*Triticum aestivum* L.) under salt stress. BMC Genom..

[8] Tibesigwa D.G., Zhuang W., Matola S.H., Zhao H., Li W., Yang L., Ren J., Liu Q., Yang J. (2025). Molecular insights into salt stress adaptation in plants. Plant Cell Environ..

[9] Chen T.H.H., Murata N. (2011). Glycinebetaine protects plants against abiotic stress: Mechanisms and biotechnological applications. Plant Cell Environ..

[10] Fitzgerald T.L., Waters D.L.E., Henry R.J. (2009). Betaine aldehyde dehydrogenase in plants. Plant Biol..

[11] Basit F., Alyafei M., Hayat F., Al-Zayadneh W., El-Keblawy A., Sulieman S., Sheteiwy M.S. (2025). Deciphering the role of glycine betaine in enhancing plant performance and defense mechanisms against environmental stresses. Front. Plant Sci..

[12] Chen J., Zhang J., Liu Y., Zhang K., Zhu F., Xie Y. (2025). Advances in the biosynthetic regulation and functional mechanisms of glycine betaine for enhancing plant stress resilience. Int. J. Mol. Sci..

[13] Holmström K.O., Somersalo S., Mandal A., Palva T.E., Welin B. (2000). Improved tolerance to salinity and low temperature in transgenic tobacco producing glycine betaine. J. Exp. Bot..

[14] Jia G.X., Zhu Z.Q., Chang F.Q., Li Y.X. (2002). Transformation of tomato with the BADH gene from *Atriplex* improves salt tolerance. Plant Cell Rep..

[15] Di H., Tian Y., Zu H., Meng X., Zeng X., Wang Z. (2015). Enhanced salinity tolerance in transgenic maize plants expressing a BADH gene from *Atriplex micrantha*. Euphytica.

[16] Yu H., Zhou X., Wang Y., Zhou S., Fu F., Li W. (2017). A betaine aldehyde dehydrogenase gene from *Ammopiptanthus nanus* enhances tolerance of *Arabidopsis* to high salt and drought stresses. Plant Growth Regul..

[17] Sun Y., Liu X., Fu L., Qin P., Li T., Ma X., Wang X. (2019). Overexpression of TaBADH increases salt tolerance in *Arabidopsis*. Can. J. Plant Sci..

[18] Ali A., Ali Q., Iqbal M.S., Nasir I.A., Wang X. (2020). Salt tolerance of potato genetically engineered with the *Atriplex canescens* BADH gene. Biol. Plant..

[19] Abid M., Gu S., Zhang Y.J., Sun S., Li Z., Bai D.F., Sun L., Qi X.-J., Zhong Y.-P., Fang J.-B. (2022). Comparative transcriptome and metabolome analysis reveal key regulatory defense networks and genes involved in enhanced salt tolerance of *Actinidia* (kiwifruit). Hortic. Res..

[20] Yu Z., Niu L., Cai Q., Wei J., Shang L., Yang X., Ma R. (2023). Improved salt-tolerance of transgenic soybean by stable over-expression of AhBADH gene from *Atriplex hortensis*. Plant Cell Rep..

[21] Tian F., Wang W., Liang C., Wang X., Wang G., Wang W. (2017). Overaccumulation of glycine betaine makes the function of the thylakoid membrane better in wheat under salt stress. Crop J..

[22] Yu M., Yu Y., Guo S., Zhang M., Li N., Zhang S., Zhou H., Wei F., Song T., Cheng J. (2022). Identification of TaBADH-A1 allele for improving drought resistance and salt tolerance in wheat (*Triticum aestivum* L.). Front. Plant Sci..

[23] Maghraby A., Alzalaty M. (2024). Genome-wide identification, characterization and evolutionary analysis of betaine aldehyde dehydrogenase (BADH), mitogen-activated protein kinase (MAPK) and sodium/hydrogen exchanger (NHX) genes in maize (*Zea mays*) under salt stress. Genet. Resour. Crop Evol..

[24] International Wheat Genome Sequencing Consortium (2018). Shifting the limits in wheat research and breeding using a fully annotated reference genome. Science.

[25] Wang M., Xia G. (2018). The landscape of molecular mechanisms for salt tolerance in wheat. Crop J..

[26] Zheng M., Li J., Zeng C., Liu X., Chu W., Lin J., Wang F., Wang W., Guo W., Xin M. (2022). Subgenome-biased expression and functional diversification of a Na+/H+ antiporter homoeologs in salt tolerance of polyploid wheat. Front. Plant Sci..

[27] Wang Y.Z., Lu J.J., Shi X.Z., Qu Y.N., Zeng L.Q., Liu X.L., Cheng X.R., Wang Y.H., Chen Z.J. (2025). Identification, characterization, and expression profiling of *Oryza sativa* betaine aldehyde dehydrogenase genes exposed to realistic environmental contamination of oxyfluorfen. Trop. Plant Biol..

[28] Islam M.S., Mohtasim M., Islam T., Ghosh A. (2022). Aldehyde dehydrogenase superfamily in sorghum: Genome-wide identification, evolution, and transcript profiling during development stages and stress conditions. BMC Plant Biol..

[29] Jia J., Deng P., Li T., Wang K., Gao L., Zhao G., Cui D., Dong Z., Li C., Zhan K. (2025). Transposable elements drive the subgenomic divergence of homoeologous genes to shape wheat domestication and improvement. Plant Commun..

[30] Jiao C., Xie X., Hao C., Chen L., Xie Y., Garg V., Zhao L., Wang Z., Zhang Y., Li T. (2025). Pan-genome bridges wheat structural variations with habitat and breeding. Nature.

[31] Zhao T., Zwaenepoel A., Xue J.Y., Kao S.M., Li Z., Schranz M.E., Van de Peer Y. (2021). Whole-genome microsynteny-based phylogeny of angiosperms. Nat. Commun..

[32] Ma L., Liu K.W., Li Z., Hsiao Y.Y., Qi Y., Fu T., Tang G.-D., Zhang D., Sun W.-H., Liu D.-K. (2023). Diploid and tetraploid genomes of *Acorus* and the evolution of monocots. Nat. Commun..

[33] Fujita Y., Fujita M., Satoh R., Maruyama K., Parvez M.M., Seki M., Hiratsu K., Ohme-Takagi M., Shinozaki K., Yamaguchi-Shinozaki K. (2005). AREB1 is a transcription activator of novel ABRE-dependent ABA signaling that enhances drought stress tolerance in *Arabidopsis*. Plant Cell.

[34] Zou Z., Khan A., Khan A., Tao Z., Zhang S., Long Q., Lin J., Luo S. (2024). Activation of ABA signaling pathway and up-regulation of salt-responsive genes confer salt stress tolerance of wheat (*Triticum aestivum* L.) seedlings. Agronomy.

[35] Yang F., Sun X., Wu G., He X., Liu W., Wang Y., Sun Q., Zhao Y., Xu D., Dai X. (2024). Genome-wide identification and expression profiling of the ABF transcription factor family in wheat (*Triticum aestivum* L.). Int. J. Mol. Sci..

[36] Hattori T., Totsuka M., Hobo T., Kagaya Y., Yamamoto-Toyoda A. (2002). Experimentally determined sequence requirement of ACGT-containing abscisic acid response element. Plant Cell Physiol..

[37] Yamaguchi-Shinozaki K., Shinozaki K. (2006). Transcriptional regulatory networks in cellular responses and tolerance to dehydration and cold stresses. Annu. Rev. Plant Biol..

[38] Seller C.A., Schroeder J.I. (2023). Distinct guard cell-specific remodeling of chromatin accessibility during abscisic acid- and CO_2_-dependent stomatal regulation. Proc. Natl. Acad. Sci. USA.

[39] Guo P., Chong L., Jiao Z., Xu R., Niu Q., Zhu Y. (2025). Salt stress activates the CDK8-AHL10-SUVH2/9 module to dynamically regulate salt tolerance in *Arabidopsis*. Nat. Commun..

[40] Wu G., Sun X., Sun Q., Kang X., Wang J., He X., Liu W., Xu D., Dai X., Ma W. (2025). Genetic variation in wheat root transcriptome responses to salinity: A comparative study of tolerant and sensitive genotypes. Int. J. Mol. Sci..

[41] Li J., Gao X., Chen X., Fan Z., Zhang Y., Wang Z., Shi J., Wang C., Zhang H., Wang L. (2023). Comparative transcriptome responses of leaf and root tissues to salt stress in wheat strains with different salinity tolerances. Front. Genet..

[42] Wang S.Z., Wang S., Li Y., Yang Y.N., Xu Y.R., Han D.J., Zeng Q.-D., Han Y.-C., Lin X.-H., Yu S.-Z. (2025). Transcriptome analysis reveals molecular mechanisms for salt tolerance in wheat (*Triticum aestivum* L.). Genomics.

[43] Yue Z., Liu Y., Zheng L., Zhang Q., Wang Y., Hao Y., Zhang M., Chen Y., Wang Z., He L. (2024). Integrated transcriptomics, metabolomics and physiological analyses reveal differential response mechanisms of wheat to cadmium and/or salinity stress. Front. Plant Sci..

[44] Wang M., Cheng J., Wu J., Chen J., Liu D., Wang C., Ma S., Guo W., Li G., Di D. (2024). Variation in TaSPL6-D confers salinity tolerance in bread wheat by activating TaHKT1;5-D while preserving yield-related traits. Nat. Genet..

[45] Shen C., Yuan J., Ou X., Ren X., Li X. (2021). Genome-wide identification of alcohol dehydrogenase (ADH) gene family under waterlogging stress in wheat (*Triticum aestivum*). PeerJ.

[46] Su P., Yan J., Li W., Wang L., Zhao J., Ma X., Li A., Wang H., Kong L. (2020). A member of wheat class III peroxidase gene family, TaPRX-2A, enhanced the tolerance of salt stress. BMC Plant Biol..

[47] Nazir R., Mandal S., Mitra S., Ghorai M., Das N., Jha N.K., Majumder M., Pandey D.K., Dey A. (2022). Clustered regularly interspaced short palindromic repeats (CRISPR)/CRISPR-associated genome-editing toolkit to enhance salt stress tolerance in rice and wheat. Physiol. Plant..

[48] Niu L., Zou J., Wang W., Wang Z., Zhang Y., Luo Z., Zhao Z., Yu L. (2024). New haplotype of salt tolerance gene TaHKT1 and evaluation of salt tolerance in wheat. Seed.

[49] Xu Y., Weng X., Jiang L., Huang Y., Wu H., Wang K., Li K., Guo X., Zhu G., Zhou G. (2024). Screening and evaluation of salt tolerant wheat germplasm based on the main morphological indices at the germination and seedling stages. Plants.

[50] Peng Z., Wang Y., Geng G., Yang R., Yang Z., Yang C., Xu R., Zhang Q., Kakar K.U., Li Z. (2022). Comparative analysis of physiological, enzymatic, and transcriptomic responses revealed mechanisms of salt tolerance and recovery in Tritipyrum. Front. Plant Sci..

[51] Li Y., Zhao S., Liu G., Li J., Siddique K.H.M., Zhao D. (2025). Agronomic traits, nutrient accumulation, and their correlations in wheat, as affected by nitrogen supply in rainfed coastal saline soils. Plants.

[52] Yates A.D., Allen J., Amode R.M., Azov A.G., Barba M., Becerra A., Bhai J., Campbell L.I., Carbajo Martinez M., Chakiachvili M. (2022). Ensembl Genomes 2022: An expanding genome resource for non-vertebrates. Nucleic Acids Res..

[53] The UniProt Consortium (2025). UniProt: The Universal Protein Knowledgebase in 2025. Nucleic Acids Res..

[54] Eddy S.R. (2011). Accelerated profile HMM searches. PLoS Comput. Biol..

[55] Blum M., Andreeva A., Florentino L.C., Chuguransky S.R., Grego T., Hobbs E., Pinto B.L., Orr A., Paysan-Lafosse T., Ponamareva I. (2025). InterPro: The protein sequence classification resource in 2025. Nucleic Acids Res..

[56] Gasteiger E., Hoogland C., Gattiker A., Duvaud S., Wilkins M.R., Appel R.D., Bairoch A., Walker J.M. (2005). Protein identification and analysis tools on the ExPASy server. The Proteomics Protocols Handbook.

[57] Chou K.C., Shen H.B. (2010). Plant-mPLoc: A top-down strategy to augment the power for predicting plant protein subcellular localization. PLoS ONE.

[58] Edgar R.C. (2004). MUSCLE: Multiple sequence alignment with high accuracy and high throughput. Nucleic Acids Res..

[59] Capella-Gutiérrez S., Silla-Martínez J.M., Gabaldón T. (2009). trimAl: A tool for automated alignment trimming in large-scale phylogenetic analyses. Bioinformatics.

[60] Wong T.K.F., Ly-Trong N., Ren H., Demotte P., Baños H., Roger A.J., Susko E., Bielow C., De Maio N., Goldman N. (2026). IQ-TREE 3: Phylogenomic inference software using complex evolutionary models. Mol. Biol. Evol..

[61] Bailey T.L., Boden M., Buske F.A., Frith M., Grant C.E., Clementi L., Ren J., Li W.W., Noble W.S. (2009). MEME Suite: Tools for motif discovery and searching. Nucleic Acids Res..

[62] Lescot M., Déhais P., Thijs G., Marchal K., Moreau Y., Van de Peer Y., Rouzé P., Rombauts S. (2002). PlantCARE, a database of plant cis-acting regulatory elements and a portal to tools for in silico analysis of promoter sequences. Nucleic Acids Res..

[63] Wang Y., Tang H., DeBarry J.D., Tan X., Li J., Wang X., Lee T.-H., Jin H., Marler B., Guo H. (2012). MCScanX: A toolkit for detection and evolutionary analysis of gene synteny and collinearity. Nucleic Acids Res..

[64] Gu Z., Gu L., Eils R., Schlesner M., Brors B. (2014). circlize implements and enhances circular visualization in R. Bioinformatics.

[65] Hao Z., Lv D., Ge Y., Shi J., Weijers D., Yu G., Chen J. (2020). RIdeogram: Drawing SVG graphics to visualize and map genome-wide data on the idiograms. PeerJ Comput. Sci..

[66] Cock P.J.A., Antao T., Chang J.T., Chapman B.A., Cox C.J., Dalke A., Friedberg I., Hamelryck T., Kauff F., Wilczynski B. (2009). Biopython: Freely available Python tools for computational molecular biology and bioinformatics. Bioinformatics.

[67] Kim D., Paggi J.M., Park C., Bennett C., Salzberg S.L. (2019). Graph-based genome alignment and genotyping with HISAT2 and HISAT-genotype. Nat. Biotechnol..

[68] Pertea M., Pertea G.M., Antonescu C.M., Chang T.C., Mendell J.T., Salzberg S.L. (2015). StringTie enables improved reconstruction of a transcriptome from RNA-seq reads. Nat. Biotechnol..

[69] Love M.I., Huber W., Anders S. (2014). Moderated estimation of fold change and dispersion for RNA-seq data with DESeq2. Genome Biol..

[70] The Gene Ontology Consortium (2019). The Gene Ontology Resource: 20 years and still GOing strong. Nucleic Acids Res..

[71] Kanehisa M., Furumichi M., Sato Y., Kawashima M., Ishiguro-Watanabe M. (2023). KEGG for taxonomy-based analysis of pathways and genomes. Nucleic Acids Res..

[72] Szklarczyk D., Nastou K., Koutrouli M., Kirsch R., Mehryary F., Hachilif R., Hu D., Peluso M.E., Huang Q., Fang T. (2025). The STRING database in 2025: Protein networks with directionality of regulation. Nucleic Acids Res..

[73] Untergasser A., Nijveen H., Rao X., Bisseling T., Geurts R., Leunissen J.A.M. (2007). Primer3Plus, an enhanced web interface to Primer3. Nucleic Acids Res..

[74] Livak K.J., Schmittgen T.D. (2001). Analysis of relative gene expression data using real-time quantitative PCR and the 2−ΔΔCT method. Methods.

