# Supplementary material for: Genome-Wide Characterization and Salt-Responsive Expression Divergence of Chromosome Group 2 and Group 6 TaBADH Genes in Wheat
Source: Plants (Basel). 2026 Aug 24;15(17):2577. doi: 10.3390/plants15172577 (PMC13567364; doi:10.3390/plants15172577)
Supplement: Supplementary file 1 [file plants-15-02577-s001.zip › Plants_Supplementary_Figures.pdf]

Supplementary Materials for "Genome-Wide Characterization and Salt-Responsive Expression Divergence of Chromosome Group 2 and Group 6 *TaBADH* Genes in Wheat"

Hua Li, Mengxue Huang, Shuxin Zhang, Xiaoyu Yang, Lingyu Pan, Sitong Wang, Wanjun Yang, Hongtu Qiu, Yemeng Zhang, Chunwang Jia, Xiu Yang

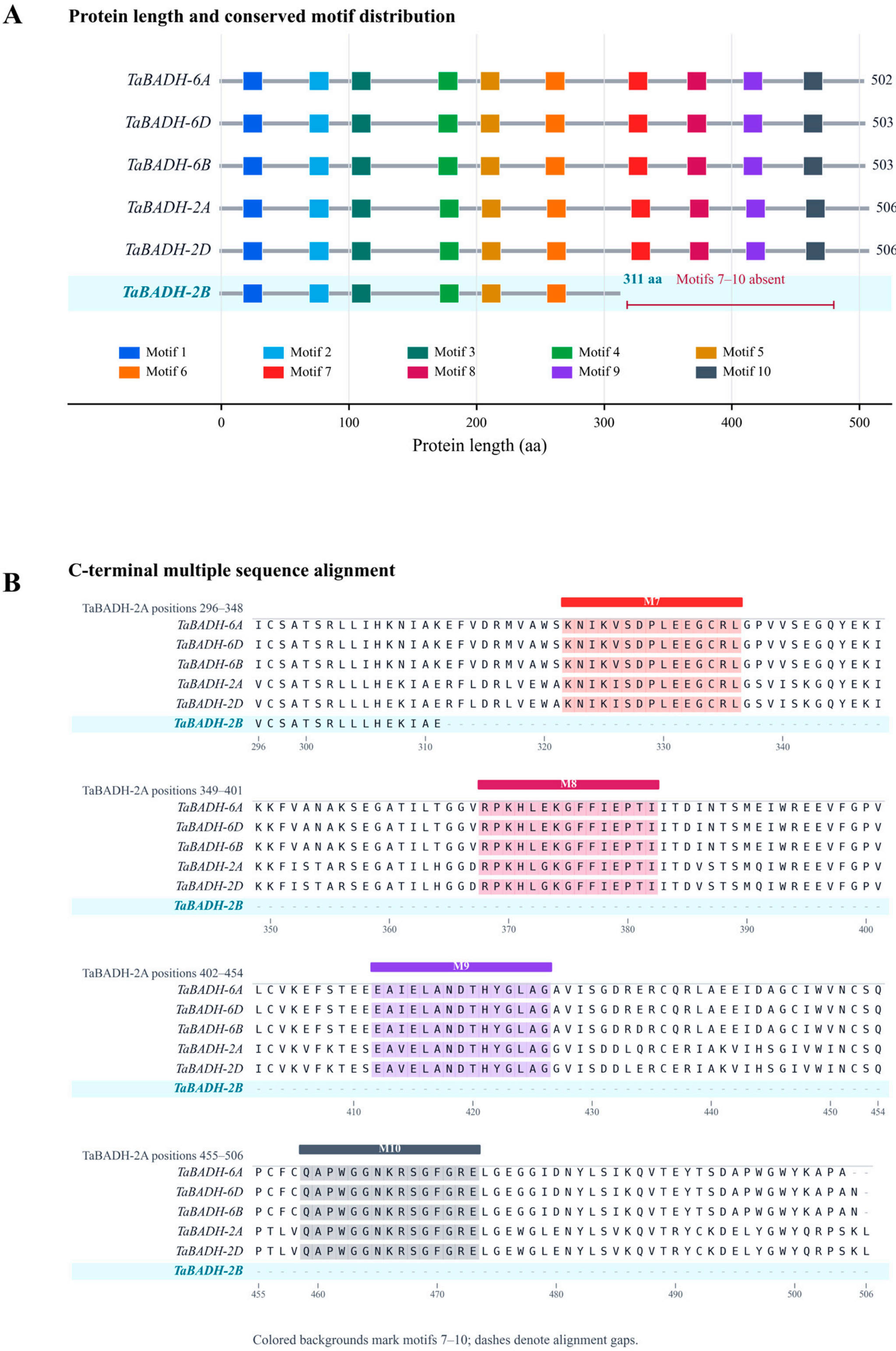

Figure S1. Structural divergence of *TaBADH-2B* relative to the other five *TaBADH* proteins, including protein lengths, conserved motif distributions and a multiple sequence alignment of the C-terminal regions corresponding to motifs 7–10.

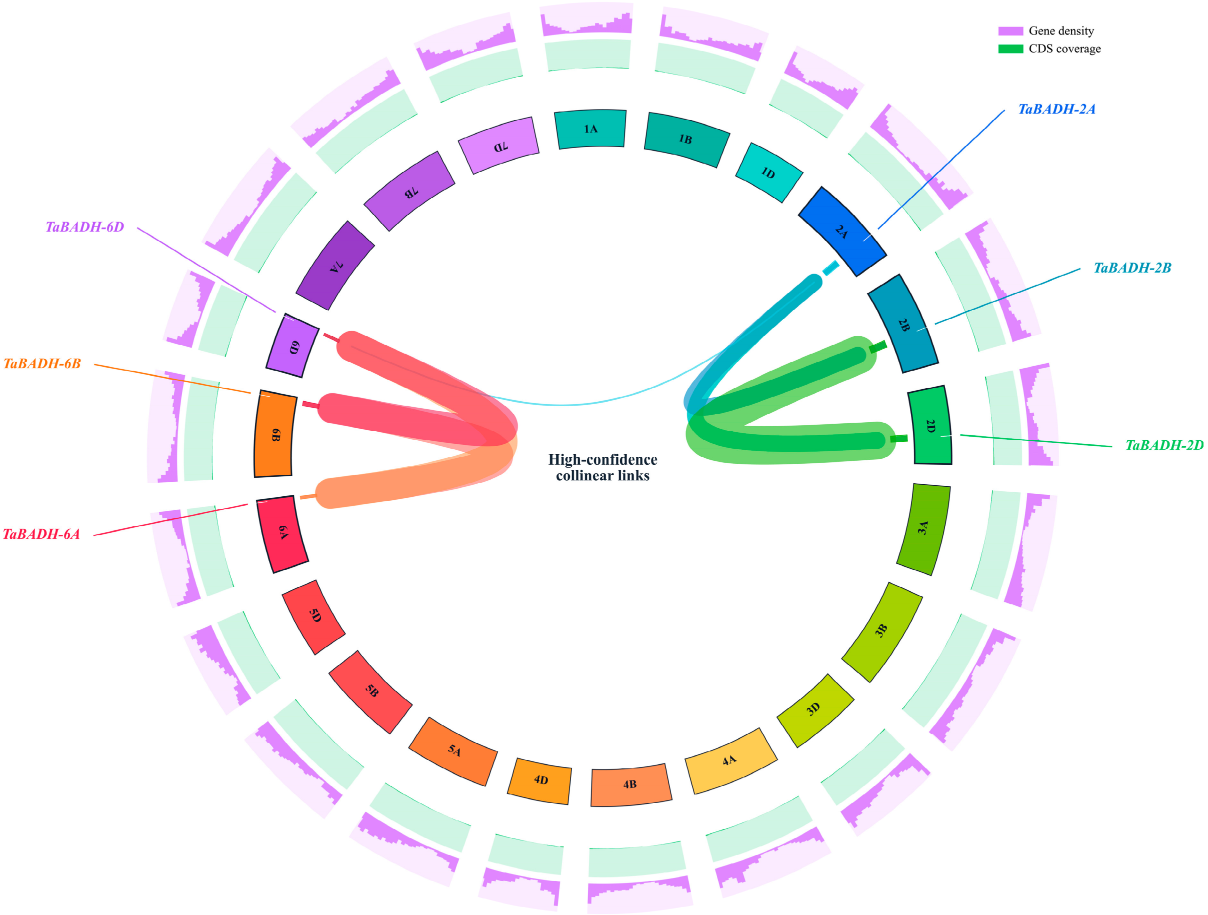

Figure S2. Intragenomic collinear relationships among the six TaBADH loci in wheat.

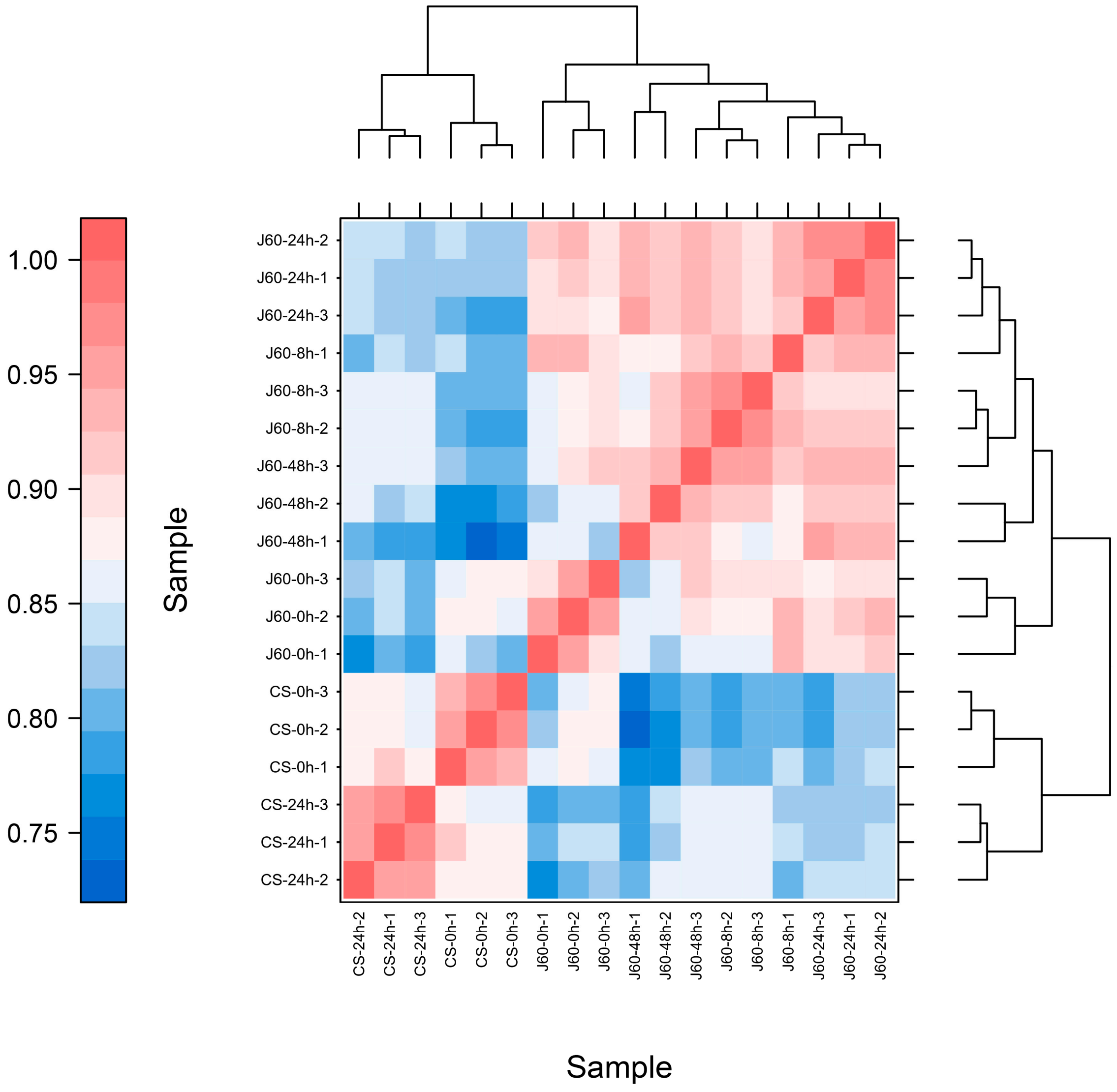

Figure S3. Pairwise Pearson correlation heatmap for the 18 RNA-seq libraries.

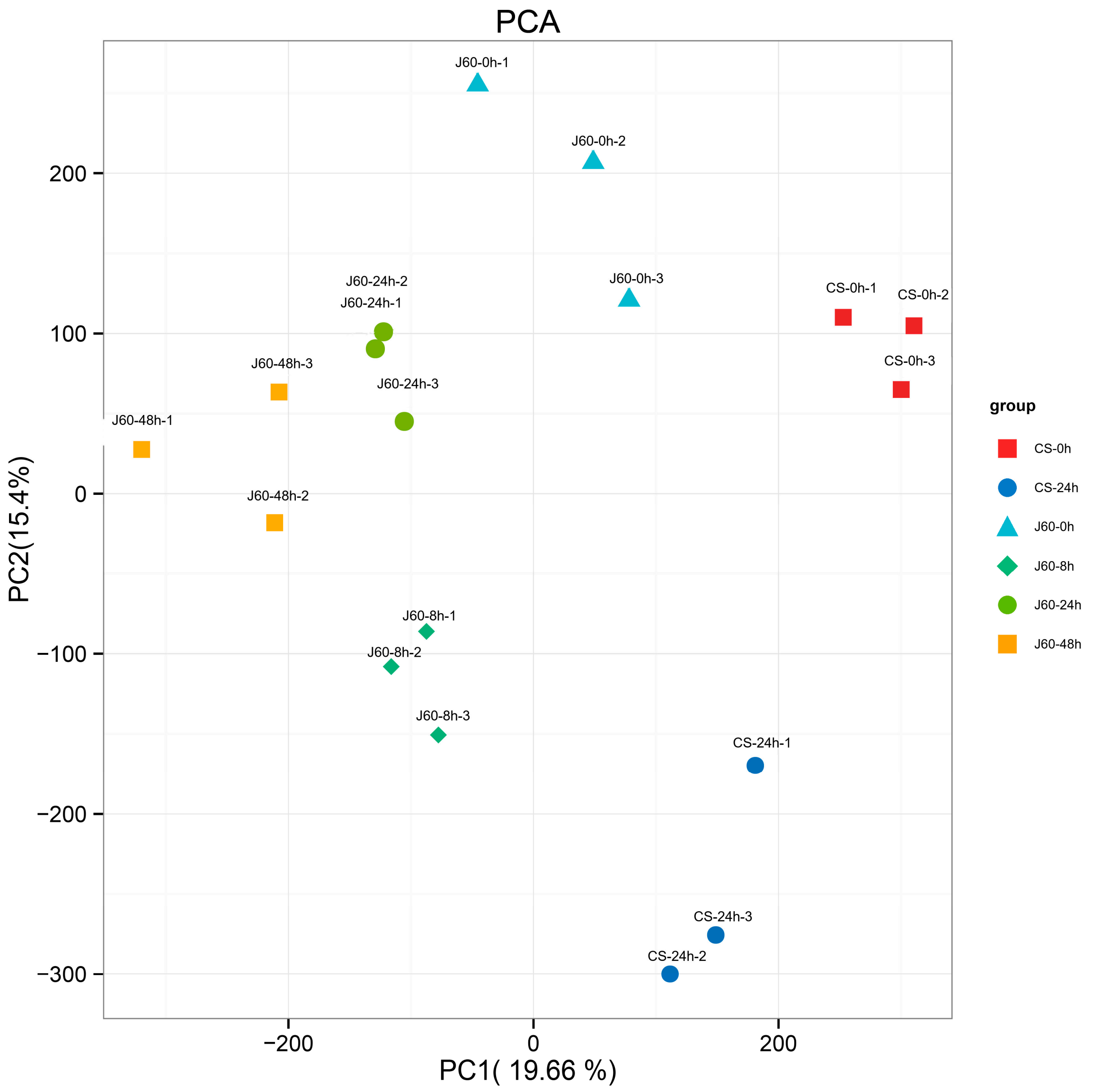

Figure S4. Principal component analysis of the 18 RNA-seq libraries**.**

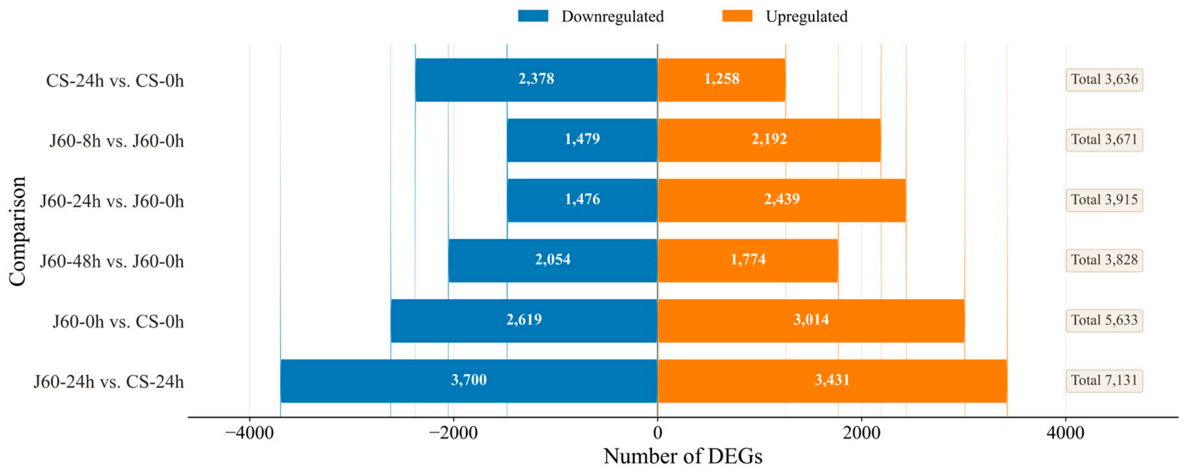

Figure S5. Numbers of up- and down-regulated genes in the six pairwise comparisons.

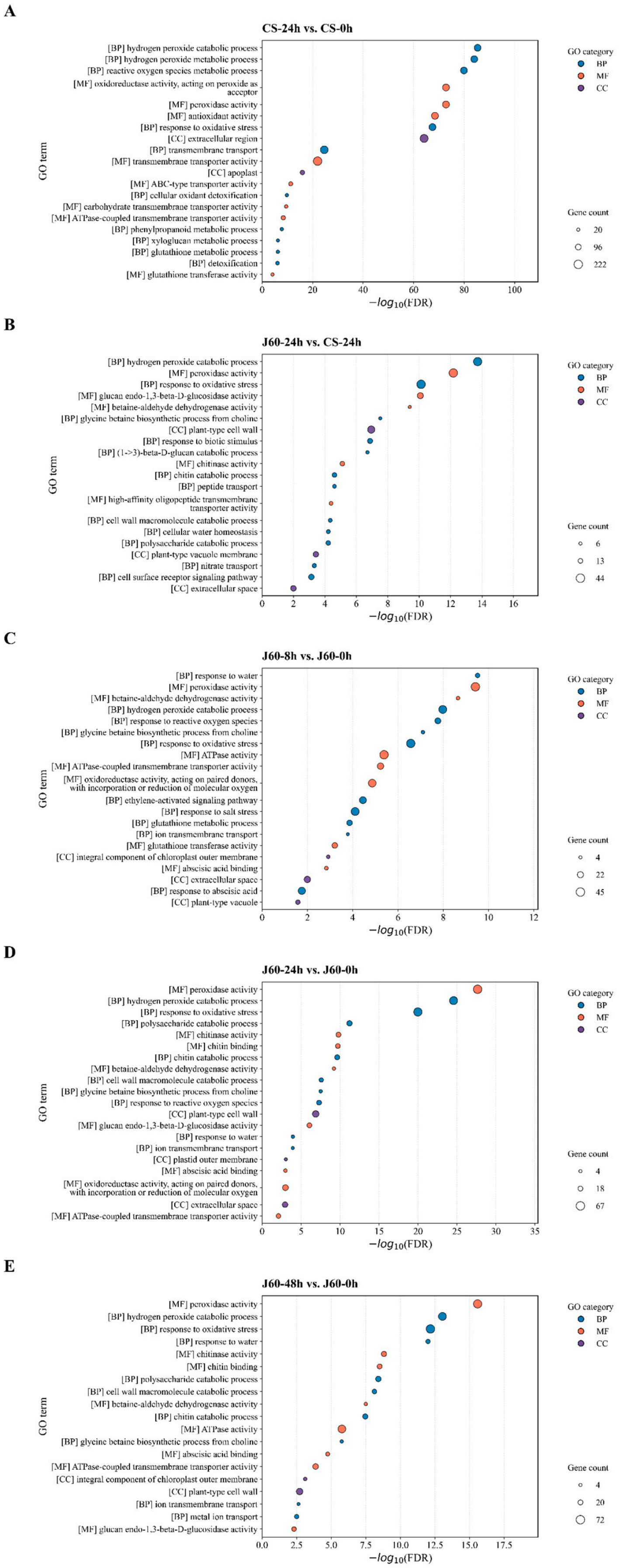

Figure S6. Complete GO enrichment results for up-regulated DEGs in five pairwise comparisons.

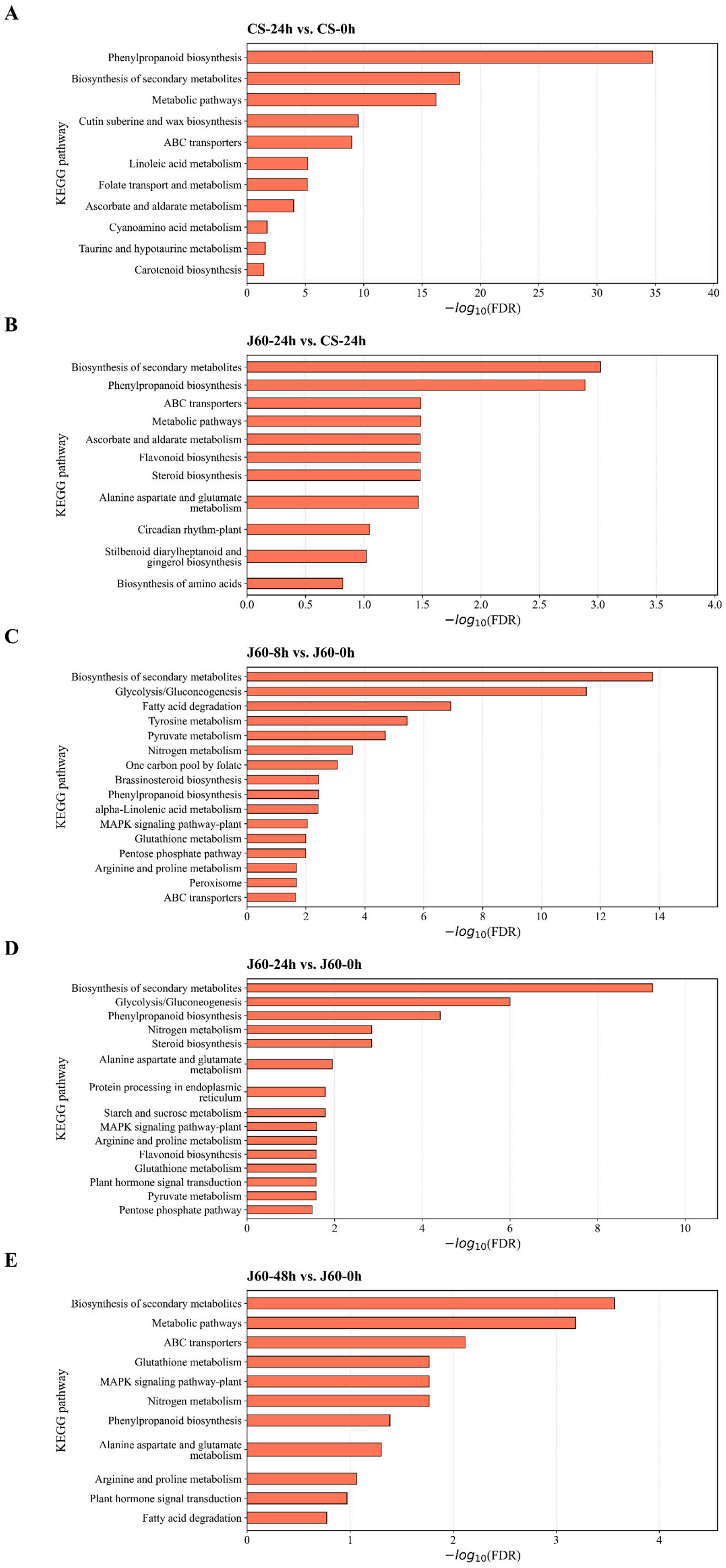

Figure S7. Complete KEGG enrichment results for up-regulated DEGs in five pairwise comparisons.

Supplementary Tables

The following supplementary tables are supplied as separate Microsoft Excel workbooks:

- Table S1. Gene-specific primers used for quantitative RT-PCR (qRT-PCR).
- Table S2. GO enrichment analysis of differentially expressed genes under NaCl treatment.
- Table S3. KEGG enrichment analysis of differentially expressed genes under NaCl treatment.
- Table S4. GO enrichment analysis of the intersection DEG set.
- Table S5. KEGG enrichment analysis of the intersection DEG set.
- Table S6. Ka, Ks and Ka/Ks values of intra-wheat *TaBADH* homologous pairs and inter-species collinear *BADH* pairs.
